# Plasmonic Enhancement of Fluorescence and Protein Dynamics in Living Mammalian Cells

**DOI:** 10.1002/adma.202501944

**Published:** 2026-04-16

**Authors:** Marco Locarno, Qiangrui Dong, Marco Post, Xin Meng, Cristiano Glessi, Nynke Marije Hettema, Nidas Brandsma, Sebbe Blokhuizen, Alejandro Castañeda Garcia, Srividya Ganapathy, Thieme Schmidt, Lars van Roemburg, Bing Xu, Chun‐Ting Cho, Liedewij Laan, Miao‐Ping Chien, Daan Brinks

**Affiliations:** ^1^ Department of Imaging Physics Delft University of Technology Delft The Netherlands; ^2^ Department of Bionanoscience Delft University of Technology Delft The Netherlands; ^3^ Department of Pediatrics and Cellular and Molecular Medicine UCSD School of Medicine San Diego California USA; ^4^ Department of Radiation Science and Technology Delft University of Technology Delft The Netherlands; ^5^ Department of Molecular Genetics Erasmus University Medical Center Rotterdam The Netherlands; ^6^ Oncode Institute Utrecht The Netherlands; ^7^ Erasmus MC Cancer Institute Rotterdam The Netherlands

**Keywords:** fluorescence, genetically encoded voltage indicators, live cells, photocycle, plasmonics, proteins, voltage imaging

## Abstract

This study shows that coupling to designed plasmonic nanoparticles can modulate the electrophysiological function of proteins in living mammalian cells. Nanostar‐shaped particles, that are robust to biological noise, are designed to enable near‐field‐coupling to plasma membrane‐localized mutated Archaerhodopsin proteins in live cells. The coupled rhodopsins exhibit enhanced fluorescence and an increased response speed to membrane voltage. Incorporating this plasmonic enhancement into a Markov chain photocycle model of the Archaerhodopsin mutant QuasAr6a, shows an increased fluorescence emission rate and manipulation of the protein dynamics through a combination of photocycle transition rate enhancements. The results show an improvement in fluorescence and voltage‐response dynamics of the functional QuasAr6a Archaerhodopsin mutant, beyond what has been achievable through genetic engineering. This opens up possibilities for engineering the biological functionality of proteins through plasmonics: manipulating protein photocycles could improve light sensitivity, change optogenetic applications, and lead to fluorescent biosensors with enhanced dynamics.

## Graphical abstract

This study demonstrates plasmonic enhancement of the function of fluorescent voltage sensing proteins (genetically encoded voltage indicators, (GEVIs), QuasAr6) in live mammalian cells. Coupling to plasmonic nanoparticles does not just increase fluorescence, but influences the protein photocycle, creating a hybrid sensor with its response speed to voltage increased beyond what has been achieved with genetic techniques.

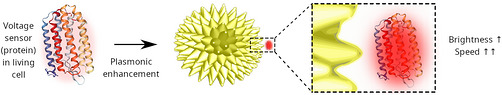

## 1 Introduction

Plasmonic nanoparticles are key components in nanophotonics [1, 2, 3, 4, 5, 6, 7, 8, 9, 10, 11] and have been used in molecular detection platforms [12, 13, 14]. The striking fluorescence enhancements [7] and altered quantum behavior [8, 15, 16, 17, 18] that can be achieved by employing plasmonic nanoparticles in single‐molecule fluorescence and SERS experiments [4] depend on the exquisite near‐field coupling between a plasmonic antenna and a fluorophore of interest. There is a large body of work on fluorescence enhancement of single biomolecules, specifically of light harvesting proteins [15, 16, 17, 19, 20, 21, 22]. Addition of plasmonic particles has also been shown to increase photocurrents in hybrid solar cells incorporating light‐harvesting II complexes, as an example of an enhancement of protein functionality in vitro [23]. Crucially, these experiments are all performed in solution or using a polymer matrix embedding.

In more complex biological environments, fluorescence enhancement of fixed cells labeled with fluorescent dyes [24] and label proteins in bacterial environments has been achieved [25, 26]. These effects have been challenging to show in live mammalian systems, where the current use of plasmonics is mostly limited to tissue level applications of plasmonic particles as contrast agents [27] or heat sources [28], due to difficulties in the detection of small molecular signals over in vivo cellular backgrounds [24, 29], and accurate placement and orientation of nanoparticles for the required coupling [30]. Nevertheless, plasmonics has been shown to be a promising tool to achieve enhanced detection of biomolecules in solution [31], and increased resolution in the optical detection of biological systems [29]. Our interest in this technology was fueled by two realizations regarding functional light‐sensitive proteins in mammals, and in particular the engineered proteins used as electrophysiological sensors and optogenetic actuators in mammalian cells: first, they often have a low fluorescence quantum yield [32, 33], since they have typically evolved to use absorbed photons for performing a light‐induced mechanical action [34]; and second, this action typically incorporates a large number of state transitions addressable through photon absorption [34, 35] or accompanied by photon emission, [36, 37]. This suggested to us that not only their fluorescence, but also their protein dynamics, and with it, their function, could be influenced through plasmonics. We set out to test the feasibility of these ideas in electrophysiologically relevant, mammalian environments.

## 2 Results

### 2.1 Calibration Using Cyanine‐5

For enhancement of transition rates, in a first‐order approximation, the resonance spectrum of the plasmonic particles should have maximum overlap with the action spectrum (absorption/emission) of the dipole emitter of interest [38]. For biocompatibility, it is particularly interesting if this spectrum is red shifted, ensuring lower phototoxicity and easier light penetration, and tunable to the dipole emitter of interest [24]. Since we are looking to use these plasmonic systems in the investigation and engineering of live mammalian cells, our plasmonic system cannot be defined by adherence to a surface for top down fabrication. We chose to use colloidal grown particles for this reason. Similarly, our system cannot be defined by a fixed distance or orientation to our molecules of interest, since the diffusive motion of nanoparticles in living biological samples, even when tethered, might lead to changes in coupling distance and relative orientation to molecular dipole emitters, hampering plasmonic enhancement [30]. We quantified these effects in simulations for different materials and geometries, and settled on gold nanostars as the optimum plasmonic system due to spectral considerations, optimized field enhancement and robustness of enhancement to positional, orientational and fabrication noise (FiguresS1– S9). To synthesize the gold nanostars, we adapted a colloidal, seed‐mediated growth protocol [39] (Figure S10).

Next, we optimized a protocol to couple plasmonic particles to fluorophores in wet lab conditions. We chose Cyanine‐5 (Cy5) as a model molecule to enhance the fluorescence because of its emission peak position and moderate quantum yield (∼30%) [40, 41].

**FIGURE 1:**
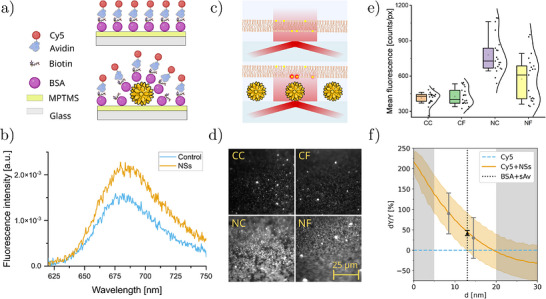
Control over nanostar‐fluorophore coupling allows tunable plasmonic enhancement. (a) Illustration of fluorescence assay on glass substrate. (b) Results from fluorescence spectroscopy in glass dishes. (c) Illustration of TIRF microscopy of a fluorescently‐lab artificial membrane without (top) and with nanostars (bottom). Not to scale. (d) Median TIRF images of the artificial membranes. Represented with the same brightness and contrast. (e) Cross comparison of the fluorescence in DOPC lipid bilayers labeled with Cy5 dye, without Fibronecton or Nanostars (CC, 410 ± 50 counts/px), with Fibronectin (CF, 430 ± 70 counts/px), with Nanostars (NC, 780 ± 160 counts/px) or with Nanostars and Fibronectin (NF, 570 ± 190 counts/px). n = 21 samples per group. Boxes represent the 25–75% range, whiskers the 10–90% range, squares the means and mid‐lines the medians. (f) Predicted mean enhancement of Cy5 (orange, solid line) within the range of uncertainty (orange band, propagated from SD), based on simulation, compared to the natural Y of Cy5 (light blue, dashed line). Data points represent the measured enhancement in panel b (mean as the black triangle, error bar propagated from noise SD) and in panel e (means as the gray dots, error bars propagated from SD). Distances for measurements from panel e are inferred by using the curve as a plasmonic ruler.

We hypothesized that Biotin–Streptavidin binding would provide a suitable way to link nanostars to fluorescent emitters under physiological conditions. Using 3‐mercaptopropyl trimethoxysilane (MPTMS, **Methods**), we immobilized gold nanostars functionalized with a complex of Bovine Serum Albumin (BSA) and biotin on glass, then added Strep‐Cy5 (Figure 1a). Fluorescence spectroscopy showed Cy5 fluorescence intensity under these conditions to be 1.49 ± 0.06 times the fluorescence in the control conditions (integrated fluorescence ± error, propagated from noise SD, Figure 1b), in agreement with the theoretical value (Equation (S7)) considering the combined lengths of streptavidin (5 nm) [42] and BSA (8 nm) [43] as a spacer larger than the quenching range [1], the already significant quantum yield of Cy5 [40, 41], and the estimated average field enhancement of a factor 1.78 from simulations (Figure S11).

To apply plasmonic enhancement in living mammalian cells, this effect needs to be compatible with live cell conditions, either in cell culturing or in model animal conditions [24]. We reasoned that first applications would focus on membrane proteins, as these are readily accessible for coupling to plasmonic particles. Given the sensitivity of the enhancement to the dielectric environment, we wondered whether the lipid bilayer cell membrane or the extracellular matrix would influence our particles enough to require adapting their design. To test this, we immobilized nanostars on glass and used Total Internal Reflection Fluorescence (TIRF) microscopy to investigate the effect on enhancement of an artificial lipid bilayer and a layer of the extracellular matrix protein fibronectin (Methods section, Figure 1c). In this in vitro experiment, we positioned lipid bilayers labeled with Cy5 on the sample and imaged the fluorescence with and without nanostars, and with and without fibronectin (Figure 1d). In the experiment where nanostars were directly coupled to Cy5, we measured a fluorescence of 1.9 ± 0.5 (mean ± error, propagated from SD) times the fluorescence of the control measurement without nanostars, while the presence of the fibronectin layer reduced this effect to 1.3 ± 0.5 (mean ± error, propagated from SD) times the fluorescence in the control experiment, probably by the fibronectin acting as a spacer (Figure 1e) [1]. In combination with the Strep‐Cy5 results (Figure 1b), we estimate the distance between the Cy5 molecules and the nanostars to be 14.5 nm with fibronectin and 8.5 nm without fibronectin (Figure 1f), for an effective 6 nm thickness of the fibronectin layer.

### 2.2 Plasmonic Enhancement of Functional Protein Fluorescence in Living Mammalian Cells

To test our system in living mammalian cells, we chose Archaerhodopsin as a model light‐sensitive protein. Several mutants of this protein exist (the genetically encoded voltage indicators (GEVIs) QuasAr1‐6a,b [33, 44, 45]; Archon1,2 [46]; NovArch [47]), which are all known for their low fluorescence quantum yield. Their emission spectrum, which was measured to be between 640 and 800 nm [32, 48], is in an interesting range for bioimaging applications. Moreover, Archaerhodopsin‐3 is a voltage‐sensitive proton pump [32, 33, 45, 49], and all of its mutants have a photocycle and fluorescence that depend on the voltage across the cell membrane in which the protein is embedded, making it an interesting proof of principle model for manipulation of protein function using plasmonics.

**FIGURE 2:**
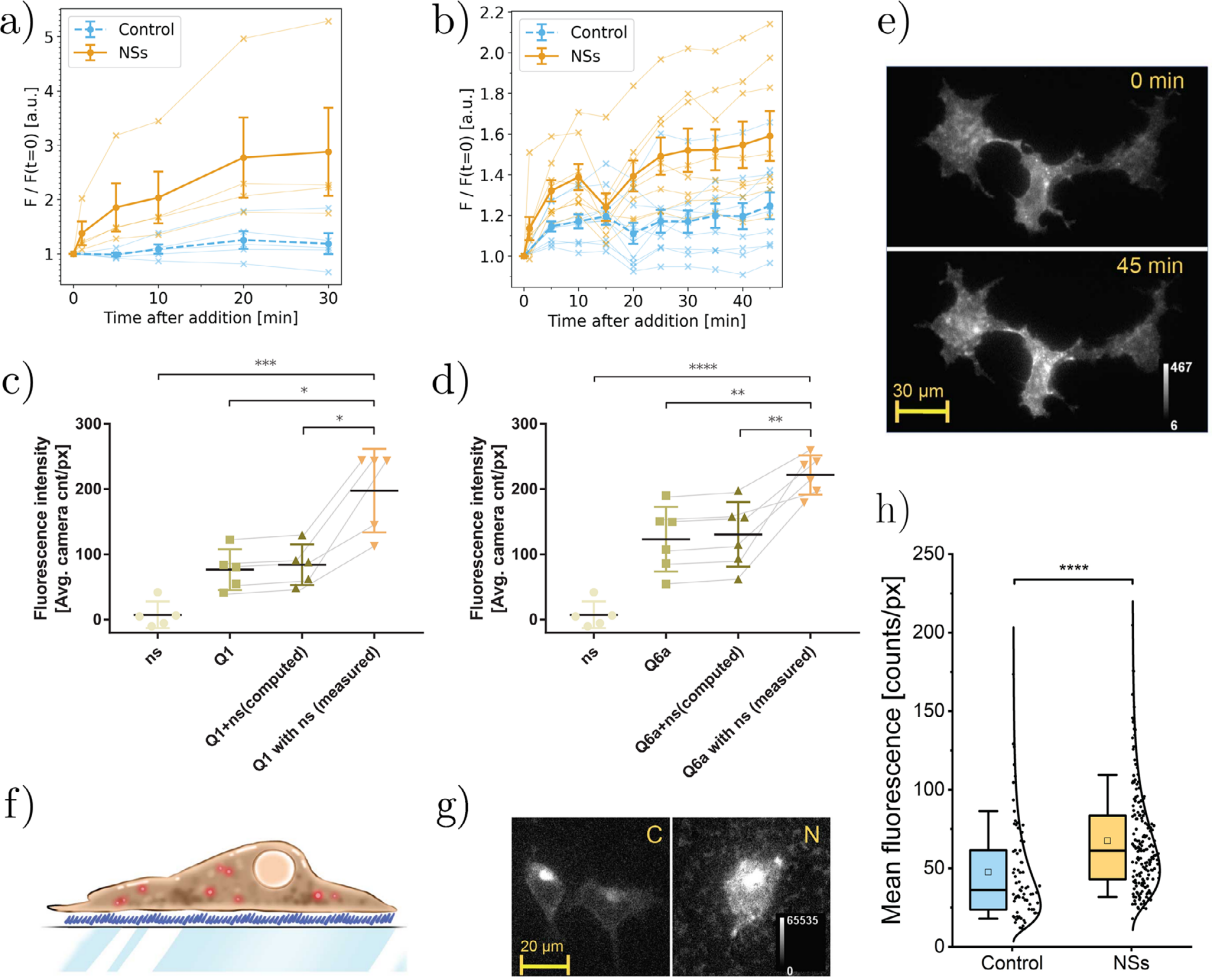
Gold nanostars enable plasmonic enhancement of QuasAr fluorescence in living cells. (a) Mean fluorescence of cells expressing QuasAr1, normalized to the first frame, after the addition 400μl of nanostars solution (NSs), compared to no addition (control). Error bars correspond to the standard error. Over time, the nanostars gradually diffuse and sediment on the cells, enhancing the fluorescence. Crosses indicate individual measurements, filled circles averages across all trials. n = 5 samples for the control group, n = 4 samples for the NSs group. (b) Mean fluorescence of cells expressing QuasAr6a, normalized to the first frame, after the addition 400μl of nanostars solution (NSs), compared to no addition (control). Error bars correspond to the standard error. Crosses indicate individual measurements, and filled circles averages across all trials. n = 10 samples for the control group, n = 8 samples for the NSs group. (c) Fluorescence analysis of QuasAr1‐expressing cells with and without resonant nanostars. Fluorescence values were processed using the same background‐subtraction procedure as in Figure 2a,b. ns denotes the nanostar‐induced fluorescence after subtraction of the signal before nanostar addition. Q1 represents the background‐subtracted fluorescence of QuasAr1‐expressing cells. Q1 + ns (computed) is the arithmetic sum of QuasAr1 fluorescence and nanostar‐only fluorescence. Q1 with ns (measured) denotes the background‐subtracted fluorescence from the same cells 20 min after nanostar addition. For QuasAr1, the measured fluorescence reached 2.61 ± 0.63‐fold above the computed Q1 + ns value (mean ± SEM). n = 5 samples per group. (d) Fluorescence analysis of QuasAr6a‐expressing cells with and without resonant nanostars, processed identically to panel **c**. n = 5 samples for the nanostars group, n = 6 for the Q6a group. Q6a + ns (computed) represents the arithmetic sum of QuasAr6a and nanostar‐only fluorescence, and Q6a with ns (measured) denotes the background‐subtracted fluorescence from the same cells 20 min after nanostar addition. For QuasAr6a, the measured fluorescence reached 1.90 ± 0.28‐fold above the computed value (mean ± SEM). (e) Example of fluorescent cells in the QuasAr6a test sample before and after the addition of nanostars. (f) Illustration of a fluorescent HEK293T cell plated on fibronectin, layered above a glass dish covered in nanostars (in blue). (g) Comparison of the median cell in the control screening (left) and in the nanostars sample (right). Individual nanostars responsible for enhancement are too small to be distinguished but nanostar aggregates are visible either as dim autofluorescence outside the cell, or spot‐like shadows under the cell. (h) Screening results show a statistical difference between a sample without (control) and with nanostars (NSs). Boxes represent the 25–75% range, whiskers the 10–90% range, squares the means and mid‐lines the medians. Uncertainty propagated from the standard error. Significance levels reported in Table S3. Statistical significance was assessed using one‐way ANOVA followed by t‐tests with Bonferroni correction. n = 73 samples for the control group, n = 180 samples for the NSs group. (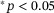, 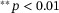, 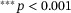, 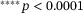).

Having shown that cell membrane embedding and a layer of the extracellular matrix protein fibronectin do not hinder plasmonic enhancement of fluorophores, we added colloidal nanostars to dishes containing HEK293T cells expressing either QuasAr1 (Figure 2a,c) or QuasAr6a (Figure 2b,d). Due to the dynamic and heterogeneous nature of the nanoparticle–cell interface, the exact spatial distribution of nanostars relative to the cell membrane cannot be determined directly in these experiments. We also cannot exclude that a fraction of the nanostars is internalized by the cells. Nanostars were added at a concentration of 5.3 × 10^10^ particles mL^−1^ (see Methods), corresponding to approximately 2 × 10^10^ particles per dish. Prior to imaging, cells were washed in protein‐free extracellular buffer (EC buffer) to minimize the presence of free proteins in solution during the measurements. We note that nanostars may acquire a protein corona upon initial exposure to culture medium, which is not explicitly characterized here, but can be considered part of the effective local biological environment in which the nanoparticles operate. We did not observe signs of acute phototoxicity or morphological degradation during the course of the experiments, as cells remain morphologically stable over tens of minutes (Figure 2e), consistent with the reported biocompatibility of gold nanoparticles under similar in vitro conditions [50].

We noticed an enhancement in the fluorescence of individual cells over time (Figure 2a). We quantified this enhancement as function of the elapsed time after addition of the nanostars and noticed fluorescence increasing with time up to 2.4 ± 0.8 (QuasAr1, mean ± error, propagated from SE, Figure 2a) and 1.3 ± 0.1 (QuasAr6a, mean ± error, propagated from SE, Figure 2b) times its original value (corrected for non‐protein‐fluorescence contributions, **Methods**). To assess the magnitude of the fluorescence increases originating from interactions between the proteins and the resonant nanostars, compared to potential contributions from nanostar autofluorescence, we performed a set of control measurements. Nanostars exhibited negligible intrinsic fluorescence when added to non‐expressing cells (Figure 2c,d). We then quantified the fluorescence from QuasAr1‐ or QuasAr6a‐expressing cells, as well as the fluorescence measured after introducing nanostars onto the same cells. The fluorescence of QuasAr1‐ and QuasAr6a‐expressing cells was 10 resp. 16 times larger than that of non‐expressing cells with nanostars; consistent with Figure 2a,b, the cellular fluorescence after nanostar addition then substantially exceeded the arithmetic sum of GEVI and nanostar fluorescence. For QuasAr1‐, respectively QuasAr6a‐expressing cells, the measured fluorescence was 2.61 ± 0.63, respectively 1.90 ± 0.28 times the value obtained by linear summation of nanostar fluorescence and protein fluorescence. The amount of extra fluorescence obtained in QuasAr1‐, resp. QuasAr6a‐expressing cells, by adding the nanostars, was 16 resp. 13 times larger than the autofluorescence of the nanostars themselves. The observation of enhanced fluorescence indicates that a sufficient fraction of nanoparticles is present in near‐field proximity to membrane‐localized proteins despite possible internalization.

We tested the difference between this addition of nanostars to cellular buffer, and embedding in the fibronectin layer. We immobilized gold nanostars on glass, coated the surface with fibronectin, and plated HEK293T cells expressing QuasAr6a (Figure 2f). As a control sample, we plated HEK293T cells expressing QuasAr6a on glass dishes containing only the fibronectin layer. We measured the fluorescence at the bottom surface of the cells (Figure 2h) and achieved in the test samples a fluorescence that was 1.69 ± 0.19 (median ± error, propagated from the SE) times that of the control sample. This enhancement value is confirmed by visualization of the fluorescence brightness of the median cell in a control sample and an enhanced sample (Figure 2g). The difference in enhancement of fluorescence caused by applying nanostars to the top surface (1.28 ± 0.12 times, Figure 2b), compared to that caused by embedding the nanostars in the fibronectin adhesion layer, (1.69 ± 0.19 times, Figure 2h) indicates that the glycocalyx [51, 52] surrounding the cells acts effectively as a spacer between the fluorophores and the nanoparticles applied to the top surface of the cells. The glycocalyx is thinner at the bottom of the cells because the formation of integrin‐fibronectin bonds induces mechanical strain [53]. We estimated an effective distance from QuasAr6a of 13.5 nm for the nanostars embedded in the fibronectin layer and 16 nm for the nanostars deposited above the cells (Equation (S1– S14), Figure 1f); we estimated the glycocalyx to cause an average of 8.5 nm larger spacing on top of the cells than on the bottom (Equation S15).

### 2.3 Plasmonic Enhancement of Protein Function in Living Mammalian Cells

**FIGURE 3:**
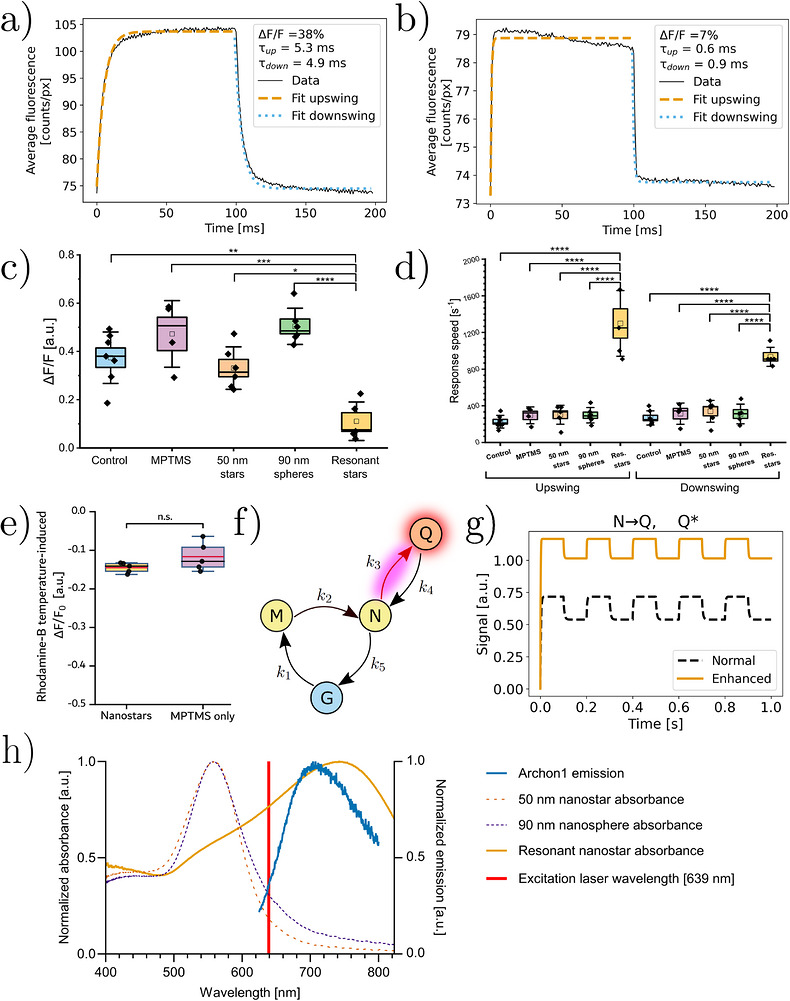
Plasmonic enhancement by gold nanostars manipulates the photocycle of QuasAr6a and modifies its response to voltage. (a) Average trace of the median control cell expressing QuasAr6a under the influence of a square wave in membrane potential (−70 to +30 mV). (b) Like a, but in the presence of nanostars. (c) Voltage sensitivity of QuasAr6a without (control) and with nanostars (Resonant stars). Controls include measurements with an MPTMS layer only (MPTMS), with 50 nm gold nanostars (50 nm stars), and 90 nm gold nanospheres (90 nm spheres). Boxes represent the standard errors, whiskers the standard deviation, squares the means and mid‐lines the medians. The mean sensitivity of the control is compatible with the value reported in the literature 0.43 ± 0.04 [45]. Under the influence of nanostars, the mean sensitivity decreases by 71% compared to the control. We only observe a change in sensitivity upon application of resonant nanostars, not in any of the other control conditions. Overall significance tested with ANOVA, significance between groups assessed with post‐hoc t‐test with Bonferroni correction, significance levels reported in Table S3. n = 5 for the control group, n = 4 for the MPTMS group, n = 6 for the 50 nm stars and 90 nm spheres groups, n = 5 for the resonant stars group. (d) Response speed of QuasAr6a without (control) and with nanostars (Resonant stars). Controls include measurements with an MPTMS layer only (MPTMS), with 50 nm gold nanostars (50 nm stars), and 90 nm gold nanospheres (90 nm spheres). Boxes represent the standard errors, whiskers the standard deviation, squares the means and mid‐lines the medians. Both the upswing and the downswing transition become faster when the proteins are coupled to the nanostars. We only observe a change in response speed to voltage changes upon application of resonant nanostars, not in any of the other control conditions. Overall significance tested with ANOVA, significance between groups assessed with post‐hoc t‐test with Bonferroni correction, significance levels reported in Table S3. Number of samples same as panel **c**. (e) Temperature measurements using Rhodamine B in wild‐type HEK293T cells plated on MPTMS‐only dishes or on dishes coated with nanostars. Cells were illuminated with a 639 nm laser in alternating 10 s ON/10 s OFF cycles. Nanostar‐coated samples showed no significant additional heating compared with controls (mean ± SEM, n = 5, Welch's t‐test, p=0.172). n = 5 samples per group. (f) Proposed photocycle for QuasAr6a; fluorescence is thought to come from the Q‐state, $k_{2}$ is voltage‐dependent and $k_{3}$ is photoactivated. (g) Numerically simulated traces for the apparent occupancy of the Q‐state of QuasAr6a under the influence of a square wave in membrane potential (−70 to +30 mV). Plasmonic enhancement is deduced to enhance $k_{3}$ and the quantum yield of the Q‐state. (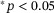, 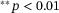, 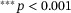, 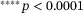) (h) Normalized extinction spectra of the resonant nanostars, 90 nm nanospheres, and 50 nm nanostars. The normalized fluorescence emission spectrum of Archon1 [48] represents the Q‐state emission band shared by Archaerhodopsin mutants. The solid red vertical line indicates the 639 nm excitation laser wavelength. Plasmonic enhancement of the photoactivated N→Q transition ($k_{3}$) requires field enhancement at the laser wavelength, while enhancement of Q‐state fluorescence requires overlap with the emission band (660–800 nm). Only the resonant nanostars provide significant enhancement in both spectral regions. Wavelength ranges for the N→Q absorption and Q‐state emission are estimated from spectroscopic studies of Arch3 derivatives [32, 48, 49].

Fluorescence enhancement of dim genetically encoded voltage indicators (GEVIs) like QuasArs is one possible application of coupling plasmonic antennas to proteins in living mammalian cells. Given that the detection kinetics of GEVIs are determined by a photocycle with potential absorption and emission events at multiple stages [34, 49, 54], we were interested in whether coupling to plasmonic antennas might also be used to enhance the function of these proteins. We performed a voltage clamp experiment on HEK293T cells expressing QuasAr6a plated on fibronectin over immobilized gold nanostars to test this. We varied the voltage across the cell membrane in steps from –70 to +30 mV and recorded the fluorescence response without (Figure 3a) and with nanostars in the fibronectin layer (Figure 3b). We noticed a decrease in voltage sensitivity from a ΔF/F of 0.37 ± 0.04 to 0.11 ± 0.04 (mean ± SEM, Figure 3c), mostly caused by the increase in baseline fluorescence, rather than a change in the absolute response to voltage. However, we noticed a strong increase in response speed from $k_{up}$ = 230 ± 70 $s^{-1}$ to $k_{up}^{\prime }$ = 1300 ± 400 $s^{-1}$ and from $k_{down}$ = 270 ± 80 $s^{-1}$ to $k_{down}^{\prime }$ = 930 ± 100 $s^{-1}$ (mean ± SD, Figure 3d).

We performed a number of control experiments to assess whether these measured effects were due to plasmonic enhancement. First, we verified that gold nanostructures did not induce measurable heating under our illumination conditions using the temperature‐sensitive dye Rhodamine B. As shown in Figure 3e and Figure S12, nanostar‐coated dishes exhibited a temperature rise indistinguishable from MPTMS‐only controls. We then examined whether QuasAr6a voltage responses were altered by different gold nanoparticles immobilized on the coverslip. All particles, including 50 nm nanostars, 90 nm nanospheres and our resonant nanostars, were deposited using the same MPTMS silanization protocol, ensuring identical surface chemistry. Across all conditions (Figure 3d), only the resonant nanostars produced a measurable enhancement in the response to voltage of QuasAr6a compared to the control. Thus, the measured effects are unlikely to be explained by a change in temperature, chemical composition of the surface layer of the dish, mechanical interaction, or electrostatic potential of the particles, but instead are consistent with plasmonic enhancement and near field‐coupling to the resonant stars. We therefore interpret the observed enhancement in fluorescence and response kinetics as a functional indication of near‐field coupling between nanostars and membrane‐localized proteins.

Following the template photocycle for Arch [49], we modeled a phenomenological photocycle for QuasAr6a as a continuous‐time finite‐state Markov chain (Figure 3f) [55]. Here, the M→N transition is voltage sensitive, the N→Q transition is photosensitive, and Q is the excitable fluorescent state. We modeled fluorescence as proportional to the Q‐state population multiplied by field enhancement and fitted our data to models with varying transition rates and field enhancement. We found the best fit to the data, including enhanced brightness, decreased voltage sensitivity, and faster kinetics, for a model incorporating $k_{3}$ = 443 $s^{-1}$ without plasmonic enhancement and $k_{3}$ = 1184 $s^{-1}$ and ${\left|\vec{E}\right|}^{2}/{\left|\vec{E^{0}}\right|}^{2}$ = 1.34 with plasmonic coupling (Figure 3g, Figure S13, complete parameters and results available in Tables S1 and S2). Enhancement of only the quantum yield evidently does not explain the change in kinetics (Figure S13a), and enhancement of only $k_{3}$ leads to a worse fit of the data (Figure S13b). The model therefore suggests that the increased fluorescence and response speed to voltage can be explained by differential enhancement of absorption of the photon promoting the N→Q transition and quantum yield of the Q‐state fluorescence, tunable by the resonance spectrum of the plasmonic antenna.

The specificity of this effect to resonant nanostars can be understood from the spectral properties of the different nanoparticles and their overlap with the protein emission (Figure 3h). The 50 nm nanostars and 90 nm nanospheres used as controls have plasmon resonances peaking both at approximately 560 nm, with negligible extinction above 630 nm. In contrast, the resonant nanostars exhibit a broad plasmon resonance peaking around 740 nm. Since the photoactivated N→Q transition ($k_{3}$) depends on absorbing a photon at the excitation laser wavelength (639 nm), and the Q‐state emission occurs between 660 and 800 nm [32, 48, 49], plasmonic enhancement of both processes requires significant enhancement in this spectral region. The control particles provide no enhancement above 630 nm, consistent with the absence of any measurable effect on fluorescence or voltage‐response kinetics in those conditions.

## 3 Discussion

In this study, we enhanced the fluorescence and voltage response speed of membrane‐localized QuasAr proteins. While plasmonic fluorescence enhancement has been extensively studied in simplified in vitro systems, the present results show that such effects can also be observed and influence protein function in living mammalian cells, where the environment is inherently more complex. We unraveled the effects on the measured plasmonic enhancement of the different components in the dielectric environment of cells and found that, while cell membrane, glycocalyx and extracellular matrix proteins do have an effect, the enhancements we measured are robust to the kinds of variability one can expect in mammalian environments. The observation of consistent effects across cells despite variability in nanoparticle positioning suggests that precise control of nanoscale geometry is not required for functional modulation. This indicates that plasmonic enhancement can operate effectively even in the heterogeneous environment of the mammalian cell membrane. Our data also show that the fluorescence enhancement arising from the interaction between the GEVIs and the resonant nanostars, is significantly larger than the minimal autofluorescence of the nanostars themselves. This indicates that the effect is not only detectable, but sufficiently strong to remain relevant even in the presence of biological background and experimental variability. Together, the robustness of the measured effects, and the negligible extra autofluorescence from the nanostars, suggests that this approach could be relatively straightforwardly applied in more complex biological settings, such as neuronal and in vivo systems. Moreover, because plasmonic enhancement is a fast physical phenomenon lasting tens of femtoseconds [9], it would be compatible with multiphoton excitation or other nonlinear imaging methods.

The applicability of this concept is helped by well‐established methods to link (gold) nanoparticles to biomolecules [28], which would allow for even more precise control of distance and orientation of particles. The dense‐star geometry shown in this work is, in this combination, the critical shape for a plasmonic antenna, due to the robustness of its coupling against positional and orientational noise Figure S1.

We report voltage responses of QuasAr6a, influenced by plasmonic enhancement, as step responses fitted to a single exponential curve. Based on probable variation in coupling strengths to the plasmonic particles of individual QuasAr6a proteins, it is reasonable to expect an inhomogeneous distribution of responses here, requiring a fit to a sum of exponentials(Supporting Information). For the sake of applicability of the assay in electrophysiological experiments, we report the values averaged per cell and with an effective response rate $k_{eff}$.

Archaerhodopsins are natural proton pumps [56]; the QuasAr and Archon mutants have been created to break the proton pumping function so that these proteins can be used as GEVIs [32, 33]. These proteins have gone through years of protein engineering efforts to increase their functionality [32, 33, 45, 47, 48, 49]. Protein evolution of GEVIs often leads to increases in brightness at the expense of the speed of responses to voltage changes [35]. Plasmonic enhancement shows a way to achieve both effects simultaneously, to an extent that has so far not been achieved by genetic engineering of the sensor. We hypothesize that a controlled trade‐off between the enhancement of absorption ($k_{3}$) and emission (Y) can be used to fine‐tune voltage sensitivity, speed, and brightness of GEVIs, through shifting of the strengths of different resonance modes in plasmonic antennas.

In conclusion, we showed plasmonic enhancement of fluorescence of fluorophores and proteins embedded in cell membranes and live mammalian cells, and demonstrated that tunable plasmonic coupling between gold nanostars and QuasAr6a modulates both the fluorescence intensity and voltage response of this optogenetic voltage sensor in living mammalian cells, amounting to an enhancement of the sensing function of these proteins by creating a faster response to voltage changes across the cell membrane. This enhancement is consistent with near‐field coupling of plasmonic particles to membrane‐localized proteins, and is dependent on the spectral overlap between the nanostar plasmon resonance and the spectra associated with the relevant state transitions in QuasAr6a. Our results indicate that plasmonic near‐field interactions can directly modulate protein photophysics. Our findings highlight the potential for plasmonic nanostructures to be harnessed to engineer the functional properties of membrane proteins in situ, paving the way for new strategies in bioengineering, such as optimizing fluorescence sensors, modulating light‐driven proteins, and controlling cellular signaling with nanoscale precision.

## 4 Methods

### 4.1 Gold Nanostar Design, Synthesis and Characterization

Resonant gold nanostars were designed, synthesized and characterized in house. Methods underlying the FDTD simulations for design, colloidal synthesis and quality control using UV/Vis spectroscopy, EDX and SEM can be found in the supplementary information. The surface chemistry of the nanostars is determined by the seed‐mediated growth synthesis and subsequent exposure to aqueous buffer conditions; no additional functionalization was applied in the experiments involving live cells. For control experiments, 50 nm gold nanourchins, 90 nm standard gold nanoparticles, and 100 nm gold nanourchins were obtained from Cytodiagnostics (GU‐50‐20, G‐90‐20, and GU‐100‐100).

### 4.2 MPTMS Surface Coating and Nanoparticle Immobilization

For the preparation of the MPTMS((3‐mercaptopropyl)trimethoxysilane)‐coated dishes, a 10% v/v MPTMS solution was prepared as follows: MPTMS (2.50 mL; Sigma–Aldrich, 175617) was combined with MilliQ water (2.50 mL), acetic acid (1.67 mL; Sigma–Aldrich, ARK2183), and pure ethanol (18.3 mL; Sigma–Aldrich, EMSURE 1.00983.1000). Prior to use, glass bottom dishes (Cellvis, D35‐14‐1.5‐N) and fragments of silicon wafers were cleaned using oxygen plasma for 1 to 2 min, and all subsequent reactions were completed within a 30‐min timeframe. To prepare the substrates for cellular imaging, 150 μL of the MPTMS solution was gently dispensed into the recessed wells of the imaging dishes. These dishes were then carefully transferred to a large glass beaker and purged with nitrogen for 30 min (or until dry). Any residual MPTMS was aspirated. The dishes were subsequently subjected to a series of rinsing steps, including one wash with 150 μL of pure ethanol in the recessed well, one wash with 2 mL of pure ethanol across the entire dish, and a final rinse with 2 mL of MilliQ water across the entire dish. To ensure dryness, the dishes were placed in a vacuum oven at 65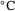 for 2 h. 200 μL of gold nanoparticles (homemade: 5.3e10 particles/mL; commercial: 3.8e9 particles/mL) were uniformly added to cover each recessed well. The dishes were then incubated overnight in a 37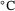 incubator, ensuring their cleanliness and contamination‐free environment. Subsequently, the nanoparticle‐coated substrates were aspirated, subjected to two water washes with 2 mL MilliQ each, and dried to near dryness. They were stored in the incubator until their intended use. For silicon substrates, the protocol remained unchanged, with volumes adjusted proportionally to the chip's surface area.

### 4.3 Fluorescence Spectroscopy Assays on Substrates

Experiments in Figure S10, Figures 1, 2 and 3 were performed with nanostars grown in‐house; the experiments in Figure 1b were performed with commercially available biotin‐functionalized nanostars (by Cytodiagnostics, catalog number GUB10K‐100‐25). Notably, the absorption peak was closely aligned to that of our in‐house synthesized nanostars. The OD of the undiluted sample was 50 at the resonance frequency, and the dilution ratio in the experiment was 1:10.

Glass coverslips underwent the MPTMS surface coating and nanoparticle immobilization, as indicated above. Approximately 60 μL of this MPTMS solution was applied to the glass coverslips. The uniform layer of gold nanostars (AuNS) was deposited onto the substrates by adding 1 mL of AuNS solution to each recessed well.

The containers housing the glass slides were then sealed and placed on an orbital shaker (100 rpm) in a dark environment for an incubation period of 18 h. On the following day, the AuNS‐coated glass slides underwent three rinses with Milli‐Q water, followed by gentle drying under a stream of nitrogen gas. To form monolayers of biotinylated bovine serum albumin (bBSA) on the substrates, bBSA solution in PBS (200 μL, 100 μg ${\mathrm{mL}}^{-1}$) was applied to the substrate surfaces and allowed to incubate for 1 h in a 37 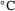 incubator. Streptavidin‐Cy5 solution (40 μL, 10 μM) was added to the substrate surfaces and incubated for 2 h in a 37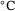 incubator. Following the incubation, the substrates were rinsed 3 times with PBS to eliminate any unbound fluorophores and gently dried using a nitrogen gas stream.

Fluorescence emission spectra of Streptavidin‐Cy5 were measured using a photomultiplier tube (R7600U‐20, Hamamatsu) coupled to a monochromator (SP2300, Princeton Instruments). Excitation light was provided by a xenon lamp connected to a monochromator (Gemini 180, HORIBA). The measurements were repeated only once for each group.

### 4.4 Supported Lipid Bilayer Formation

A glass slide covered by a silicone gasket with four 100 μL wells was prepared with different MPTMS‐immobilized nanostars and fibronectin conditions prior to the bilayer formation. The two Avanti Polar lipids (Birmingham, AL) used were 1,2‐dioleoyl‐sn‐glycero‐3‐phosphocholine (DOPC) and 1,2‐dioleoyl‐sn‐glycero‐3‐phosphocholine‐N‐(Cyanine 5) (DOPC‐Cy5). Lipids in chloroform were mixed in a molar ratio of 99.5:0.5 DOPC:DOPC‐Cy5 in a glass vial. The chloroform was evaporated with nitrogen and dried in a vacuum desiccator overnight. The dried lipid film was resuspended in 1X PBS to get a total lipid concentration of 6.5 mM. The glass vial with the solution was then vortexed in bursts of 30 s with 1‐min intervals to resuspend all lipids and to stimulate the self‐assembly of multilamellar vesicles. The solution was then sonicated in a high‐capacity bath sonicator (10% amplitude, 10 s on, 10 s off, for one hour) to get a uniform size distribution of liposomes. 100 μL of liposome solution was then deposited in each of the wells of the silicone gasket. The glass slides with liposomes were incubated for 30 min at room temperature to allow for supported lipid bilayer formation. All wells were washed four times with 200 μL PBS after incubation.

### 4.5 TIRF Microscopy

For the TIRF microscopy, an inverted microscope (NikonTi2‐E, motorized) equipped with a 100× oil immersion objective (Nikon Apo TIRF 1.49 NA) and an Andor iXon Ultra 897 camera was used. The microscope was upgraded with an azimuthal TIRF/FRAP illumination module (GATACA systems iLAS 2). Samples were exited at 642 nm at 10% laser power (GATACA CW laser, 642nm/110mW). The emission was filtered with a 706/90 filter. Samples were exited for 200 ms for each frame, and a multiplication gain 50 was used. The penetration depth automatically estimated by the system is 106 nm. Every group consists of 21 distinct samples.

### 4.6 HEK293T Culture, Lentivirus Preparation and Transduction

HEK293T cells (Catalog number ATCC CRL‐3216) with passage number <30 were cultured in Dulbecco's Modified Eagle Medium (DMEM; Gibco) supplemented with Fetal Bovine Serum (10%; VWR Seradigm Premium Grade), Penicillin (50 units/mL; Life Technologies), Streptomycin (50 μg/mL; Life Technologies), and GlutaMAX (1%, Life Technologies) at 37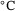 under 10% ${\mathrm{CO}}_{2}$.

For lentivirus production, HEK293T cells were seeded at 4$\cdot 10^{6}$ cells per 100 mm polystyrene dish (Sarstedt) 1 day before transfection in order to achieve 80% confluency at the moment of transfection. Cells were co‐transfected with transfer plasmid (1.5 μg) carrying either QuasAr1 or QuasAr6a, pMDLg/RRE (5 μg; Addgene plasmid #12251), pRSV/Rev (5 μg; Addgene plasmid #12253), and pMD2.G (3.5 μg; Addgene plasmid #12259) mixed in pre‐warmed opti‐MEM (400 μL) with polyethylenimine solution (60 μL, 1 μg μL 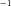 in PBS). 8 h post‐transfection, medium was replaced with 10 mL fresh cell culture medium including HEPES (25 mM). Lentivirus‐containing supernatant was collected 48 h post‐transfection after which another 5 mL fresh cell culture medium including 25 mM HEPES was added to cells. Supernatant collected 48 h post‐transfection was stored overnight at 4 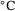 before pooling it with lentivirus collected 72 h post‐transfection. The resulting 15 mL lentivirus‐containing supernatant was filtered through 0.22 μm PVDF filters. Lentivirus was concentrated using LentiX concentrator reagent (Takara), resuspended in 1 mL PBS for a 15‐fold concentration, and stored at –80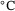 in 100 μL aliquots.

For lentiviral transduction, 2.5 × 10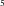 cells were seeded per 35 mm polystyrene dish (Sarstedt) 1 day before transduction in order to achieve 50% confluency at the moment of transduction. Lentiviral aliquots were rapidly thawed in a 37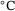 water bath. 100 μL of concentrated virus(3715 ng/mL) was added per dish. Cells were incubated with lentivirus for 24 h, after which the media was replaced with fresh cell culture medium.

Before the fluorescence and patch‐clamp measurements shown in Figures 2, 3 and Figure S12, cells were washed gently three times with 2 mL of pre‐warmed extracellular buffer (EC buffer). Cells were immersed in 2 mL of EC buffer throughout the measurement procedures. The constitution of EC buffer is 125 mM NaCl, 2.5 mM KCl, 3 mM CaCl2, 1 mM MgCl2, 15 mM HEPES, and 30 mM glucose [57]. pH of EC buffer is adjusted to 7.3 with NaOH. Osmolarity of EC buffer is adjusted to 300 mOsm with sucrose.

### 4.7 Widefield Fluorescence Microscopy

A custom‐built [58] widefield microscope was employed to investigate the fluorescence of the QuasArs expressed in cells. Three continuous wave laser sources (MLL‐FN‐639 by CNI, MLL‐III‐532 by CNI and OBIS 488 LX, by Coherent) were made uniform in polarization and beam diameter through zero‐order half‐wave plates (WPH05M‐488, WPH05M‐532 and WPH05M‐633 by Thorlabs), polarizers (CCM5‐PBS201/M by Thorlabs) and individual collimators (AC254 mounted achromatic doublets by Thorlabs). The beams were then combined using dichroic mirrors (DMLP505 and DMLP605 by Thorlabs) and led through an acousto–optic tunable filter (AOTFnC‐VIS by AA Optoelectronics) with a modulation rate of 22 kHz. With the exception of the patch clamp voltage imaging experiments, the beam diameter was expanded with a telescope (AC254 mounted achromatic doublets by Thorlabs) using flip mounts (TRF90/M by Thorlabs). The light was reflected from a Digital Micromirror Device (DMD, Vialux V7001 by Texas Instruments) into a tube lens (TTL200 by Thorlabs) and projected by the objective (Olympus

XLPLN25XWMP2, NA 1.05, working distance 2 mm) onto the sample. The emitted fluorescence was reflected by a dichroic mirror (Di03‐R405/488/532/635‐t3‐32 × 44 by Semrock), collected using a tube lens (TTL200 by Thorlabs) and filtered further depending on the emission range (FF01560/94‐25, FF01‐582/64‐25, LP02‐664RU‐25 and FF01‐790/SP‐25 by Semrock), before reaching the sCMOS camera (ORCA Flash4.0 V3, 2048 × 2048 pixels, 6.5 μm pixel size by Hamamatsu) where the image was captured in a low‐noise configuration with a total magnification of 27.8.

The maximum powers after the objective in the telescope configuration are 45 mW for the 639 nm laser and 9 mW for the 488 nm laser; without the telescope, these powers are 145 mW for the 639 nm laser and 23 mW for the 488 nm laser.

In Figure 2a the sample size is n = 5 cells for the control and n = 4 cells for the nanostar sample. In Figure 2b the sample size is n = 10 cells for the control and n = 8 cells for the nanostar sample. In Figure 2c the sample size is n = 5 cells for the control and n = 5 cells for the QuasAr1 sample. In Figure 2d the sample size is n = 5 cells for the control and n = 6 cells for the QuasAr6a sample. In Figure 2h the sample size is n = 73 cells for the control and n = 180 cells for the nanostar sample.

The statistical test used for Figure 2h is a one‐sided Mann‐Whitney test that does not assume a Gaussian distribution of the data points.

Background was subtracted in all experiments shown in Figures 2 and 3 to isolate the biomolecular emission from the auto fluorescence of the medium and particle emission. All reported values are averaged over the cellular surface area and are corrected for potential auto‐fluorescence contributions of the nanostars by subtracting, for each data point, a background fluorescence value measured away from the fluorescent cells at the same time point in the same sample (i.e. with the same distribution and settling of nanostars, the same surface functionalization, the same setup auto‐fluorescence and the same laser bleedthrough). We use a frame‐wise local background correction to minimize potential bias from heterogeneous nanostar distribution. Specifically, for each field of view, the background is defined as the median intensity of non‐cellular regions in each frame, which is then subtracted on a per‐frame basis. This method avoids artifacts introduced by uneven nanostar decoration while maintaining a consistent normalization across all conditions. The data therefore only contain contributions of (non‐)enhanced protein fluorescence, with all linearly additive contributions to the measured fluorescence factored out.

For the control experiments in Figure 2c,d, a similar procedure was followed, except a separate measurement was done with cells expressing no proteins and cells expressing QuasAr1 and QuasAr6a. Only for these measurements, an overall background value was obtained by measuring the fluorescence of non‐expressing cells before nanostar addition. Nanostars were then added to these cells and subsequent measurements were done to obtain a raw measurement of nanostar autofluorescence, from which this background value was then subtracted to arrive at the pure nanostar autofluorescence. For the measurements on QuasAr1‐ and QuasAr6A‐expressing cells in Figure 2c,d, the same procedure was followed. Fluorescence was measured before nanostar addition, and following nanostar addition. The previously obtained average background (measured on non‐expressing cells) was subtracted, to obtain the protein fluorescence before addition of nanostars, and the fluorescence of the proteins with nanostars added. (Note that another way of background subtraction here could have consisted of measuring a background away from the protein‐expressing cells before nanostar addition and subtracting this from the cellular fluorescence both before and after nanostar addition; we verified that this did not meaningfully change the reported values and elected to use the same background for all measurements in Figure 2c,d). The measurements labeled “Q1/Q6a with ns (measured)” in Figure 2c,d are therefore the only ones in the manuscript that encompass both the enhanced protein fluorescence and the autofluorescence contributions of the nanostars. These measured values are then compared to fluorescence values of the same protein‐expressing cells before nanostars were added (labeled “Q1/Q6a” in Figure 2c,d), and to those values with the average nanostar fluorescence value computationally added (labeled “Q1/Q6a+ns (computed)” in Figure 2c,d).

### 4.8 Temperature Control Experiment Using Rhodamine B

To assess potential local heating effects induced by nanostars under 639 nm illumination, we performed a control experiment using the temperature‐sensitive fluorescent dye Rhodamine B (Sigma–Aldrich, R6626). Wild‐type HEK293T cells not expressing QuasAr6a were plated on glass‐bottom dishes either coated with resonant nanostars or with MPTMS only as control.

Cells were incubated with 50 μM Rhodamine B (dissolved in Milli‐Q) for 5 min at 37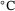, followed by three washes with EC buffer, after which cells were immersed in 2 mL of EC buffer throughout the imaging procedure. Cells were illuminated alternately with a 639 nm laser (10 s ON/10 s OFF cycles, repeated three times) to evaluate fluorescence dynamics under heating and recovery conditions. To correct for photobleaching, fluorescence intensities were compared between the last frames of the illumination period (“hot” condition) and the first frames immediately after the laser was turned back on (“cool” condition). During the OFF period, no photobleaching occurs; therefore, any fluorescence difference between “hot” and “cool” phases would indicate temperature‐dependent changes in Rhodamine B fluorescence.

For each field of view, cell regions were segmented from the first frame using Otsu thresholding to create a binary cell mask, and the background was estimated as the median pixel value outside the cell mask in each frame and subtracted from all pixels. Fluorescence traces were then normalized to the first frame. Mean ± SEM across 10 recordings was calculated for each timepoint and plotted as a fluorescence trace (Figure S12). For quantitative comparison (Figure 3e), the mean fluorescence during the “hot” and “cool” periods was determined, and the relative fluorescence decrease was calculated as:

$$\Delta F/F_{\mathrm{cool}}=\frac{F_{\mathrm{hot}}-F_{\mathrm{cool}}}{F_{\mathrm{cool}}}$$

The resulting values were compared between nanostar and control samples using Welch's unpaired t‐test.

### 4.9 Whole‐Cell Patch‐Clamp Recordings

Patch‐clamp and imaging were performed 72 h after lentiviral transduction. Transduced cells were seeded in the 14 mm glass well of fibronectin‐coated glass bottom dishes (Cellvis) at a density of 1.5×10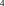 cells per well 24 h before the patch‐clamp experiment. Before patch‐clamp procedure, cells were washed gently three times with 2 mL of pre‐warmed extracellular buffer (EC buffer). Cells were immersed in 2 mL of EC buffer throughout the patch clamp procedure.

Borosilicate glass micropipettes were pulled from capillaries (BF150‐86‐10, Sutter Instrument) and filled with 10 μL of intracellular medium (IC medium). The constitution of IC medium is 125 mM K‐gluconate, 8 mM NaCl, 0.6 mM MgCl2, 0.1 mM CaCl2, 1 mM EGTA, 10 mM HEPES, 4 mM Mg‐ATP and 0.4 mM Na‐GTP, with pH adjusted to 7.3 with KOH [59]. Osmolarity of IC medium is adjusted to 295 mOsm with sucrose. Tip resistance was verified to be between 5 and 10 MΩ. Cellular responses were evoked through voltage steps ranging from –70 to +30 mV at a frequency of 5 Hz, while the fluorescence of QuasAr1 and QuasAr6a was excited at 639 nm. The camera frame rate in this setting is 1000 fps.

The statistical tests used for Figure 3c,d are one‐sided t‐tests.

### 4.10 Photocycle Modeling

The photocycle of QuasAr6a has been modeled as a 4‐state continuous‐time Markov chain, where the putative biological macrostates corresponded directly to the mathematical states. Transitions were considered unidirectional. The Markov chain was expressed as a system of linear ODEs and solved numerically in Python using the LSODA solver (Livermore Solver for Ordinary Differential equations with Automatic method switching for stiff and non‐stiff problems). The features of simulated traces (brightness enhancement, voltage sensitivities, response speeds) are compared to those of the measured traces and improved following a differential evolution algorithm. The software outputs the best parameters after a maximum of 1000 iterations, unless a plateau is reached earlier. To quantify the goodness of the fit in different conditions, we employed a Huber loss calculated on the fitted features compared to the one from the simulations. A lower loss indicates a better agreement between the simulations and the data. The fitted values for the models can be consulted in the Supplementary Information, while the code can be accessed on GitHub [60].

### 4.11 AI Methods

Various AI‐based tools were used to aid in the creation of this manuscript.

ChatGPT (GPT‐3.5, GPT‐4o and GPT‐5.1) was used to speed up and optimize the coding process in Python. All AI‐generated code was debugged and validated.

Paperpal, Typeset.io and Research Rabbit were used to assist in finding papers relevant to the current manuscript for the literature review. ChatGPT was used to check consistency between the manuscript text and cited references. As with any other library‐building tool, all suggested papers were reviewed manually regarding their relevance and accuracy prior to the decision to cite a reference in the manuscript.

ChatGPT was used to check flow of paragraphs and phrasings of specific sentences in a first draft of this manuscript, before complete revision by authors. Grammarly was used for grammar checks, correcting errors and enhancing clarity of the text. Suggestions were considered critically, especially if changes in punctuation or syntax could distort the original message conveyed in the text.

Any intellectual contributions, final decisions on content and structuring of the work, and writing of the final manuscript were exclusively made and done by credited authors.

## Author Contributions

Marco Locarno and Qiangrui Dong share first authorship. **Marco Locarno**: conceptualization, methodology, formal analysis, software, investigation, validation, resources, data curation, visualization, writing – original draft, writing – review and editing, funding acquisition **Qiangrui Dong**: conceptualization, methodology, formal analysis, investigation, validation, resources, data curation, visualization, writing – original draft, writing – review and editing, funding acquisition. **Marco Post**: conceptualization, formal analysis, investigation, validation, resources, data curation, visualization, writing – original draft, writing – review and editing. **Xin Meng**: methodology, software, investigation, validation, resources, data curation, writing – review and editing. **Cristiano Glessi**: conceptualization, investigation, validation, resources, data curation, writing – original draft, writing – review and editing. **Nynke Marije Hettema**: methodology, investigation, validation, resources, data curation, writing – original draft, writing – review and editing. **Nidas Brandsma**: methodology, formal analysis, software, investigation, validation, data curation, writing – review and editing. **Sebbe Blokhuizen**: formal analysis, software, investigation, data curation, writing – review and editing. **Alejandro Castañeda Garcia**: methodology, formal analysis, software, data curation, writing – original draft, writing – review and editing. **Srividya Ganapathy**: methodology, resources, writing – review and editing. **Thieme Schmidt**: methodology, investigation, validation, writing – review and editing. **Lars van Roemburg**: methodology, investigation, validation, writing – review and editing. **Bing Xu**: investigation, validation, resources, writing – review and editing. **Chun‐Ting Cho**: investigation, validation, resources, writing – review and editing. **Liedewij Laan**: resources, writing – review and editing, supervision. **Miao‐Ping Chien**: methodology, resources, writing – review and editing, supervision. **Daan Brinks**: conceptualization, methodology, software, validation, resources, data curation, writing – original draft, writing – review and editing, project administration, supervision, funding acquisition.

## Ethics Statement

The research in this paper does not involve local researchers. All biological material was obtained according to the Nagoya protocol.

## Conflicts of Interest

The authors declare no conflicts of interest.

## Supporting information


**Supporting File**: adma73044‐sup‐0001‐SuppMat.pdf.

## Data Availability

All data underlying manuscript figures have been uploaded to the 4TU Respository and are available at DOI: 10.4121/909b7170‐816f‐4d81‐a8b0‐12b113f29207. The data that support the findings of this study are openly available in [4TU repository4TU repository4TU repository] at [https://doi.org/10.4121/909b7170‐816f‐4d81‐a8b0‐12b113f29207], reference number [1].
