# Supplementary material for: Plasmonic Enhancement of Fluorescence and Protein Dynamics in Living Mammalian Cells
Source: Adv Mater. 2026 Apr 16;38(45):e01944. doi: 10.1002/adma.202501944 (PMC13472017; doi:10.1002/adma.202501944)
Supplement: Supplementary file 1 [file ADMA-38-e01944-s001.pdf]

PLASMONIC ENHANCEMENT OF FLUORESCENCE AND PROTEIN DYNAMICS IN LIVING MAMMALIAN CELLS

Marco Locarno,[*,†,‡] Qiangrui Dong,[†,‡] Marco Post,[‡] Xin Meng,[‡] Cristiano Glessi,[‡] Nynke Marije Hettema,[¶] Nidas Brandsma,[‡] Sebbe Blokhuizen,[‡] Alejandro Castañeda Garcia,[‡] Srividya Ganapathy,[‡,§] Thieme Schmidt,[‡] Lars van Roemburg,[‡] Bing Xu,[‖] Chun-Ting Cho,[‖] Liedewij Laan,[¶] Miao-Ping Chien,[⊥,#,@] and Daan Brinks[*,‡,⊥]

†*These authors share first authorship.*

‡*Department of Imaging Physics, Delft University of Technology, Delft, The Netherlands*

¶*Department of Bionanoscience, Delft University of Technology, Delft, The Netherlands*

§*Department of Pediatrics and Cellular and Molecular Medicine, UCSD School of Medicine, San Diego, California, United States*

‖*Department of Radiation Science and Technology, Delft University of Technology, Delft, The Netherlands*

⊥*Department of Molecular Genetics, Erasmus University Medical Center, Rotterdam, The Netherlands*

#*Oncode Institute, Utrecht, The Netherlands*

@*Erasmus MC Cancer Institute, Rotterdam, The Netherlands.*

E-mail: m.locarno@uu.nl; d.brinks@tudelft.nl

Supplementary Information

FDTD simulations

Summary

In the experiments described in this paper we use colloidal grown gold nanostars. Top down fabrication is not the method of choice for our plasmonic system, since we are looking to use this in the investigation of live mammalian cell dynamics, which means our plasmonic system cannot be defined by adherence to a surface. Similarly, our system cannot be defined by a fixed distance or orientation to our molecules of interest, since the diffusive motion of nanoparticles in living biological samples, even using molecular tethers, might lead to changes in coupling distance and relative orientation to molecular dipole emitters, hampering plasmonic enhancement.[1] We quantified these effects in simulations for different materials and geometries, (**Figures S1-S8**) and settled on gold nanostars as the optimum plasmonic system due to spectral considerations, optimized field enhancement and robustness of enhancement to positional, orientational and fabrication noise. **Figure S1** summarizes our findings for our optimized geometry, and shows a robustness of the enhancement of gold nanostars to positional and fabrication noise.

Numerical Methods

Finite-difference time-domain (FDTD) numerical simulations were conducted using Lumerical's FDTD Solutions, with scripts available on GitHub.[2] Simulations of gold nanospheres in **Figure S2** involved varying the radius R of a gold sphere submerged in water, with a focus on assessing the average field enhancement $|E|^2/|E^0|^2$ within a small cubic volume ($\Delta x = \Delta y = \Delta z = R/2$) located 4 nm from the surface. Simulations covered a range of radii ($R_{\text{min}} = 6$ nm to $R_{\text{max}} = 76$ nm) with 2 nm steps.

Gold nanostar models were crafted based on SEM micrographs of synthesized nanoparticles. To generate these models, Blender software was employed to create complex meshes.

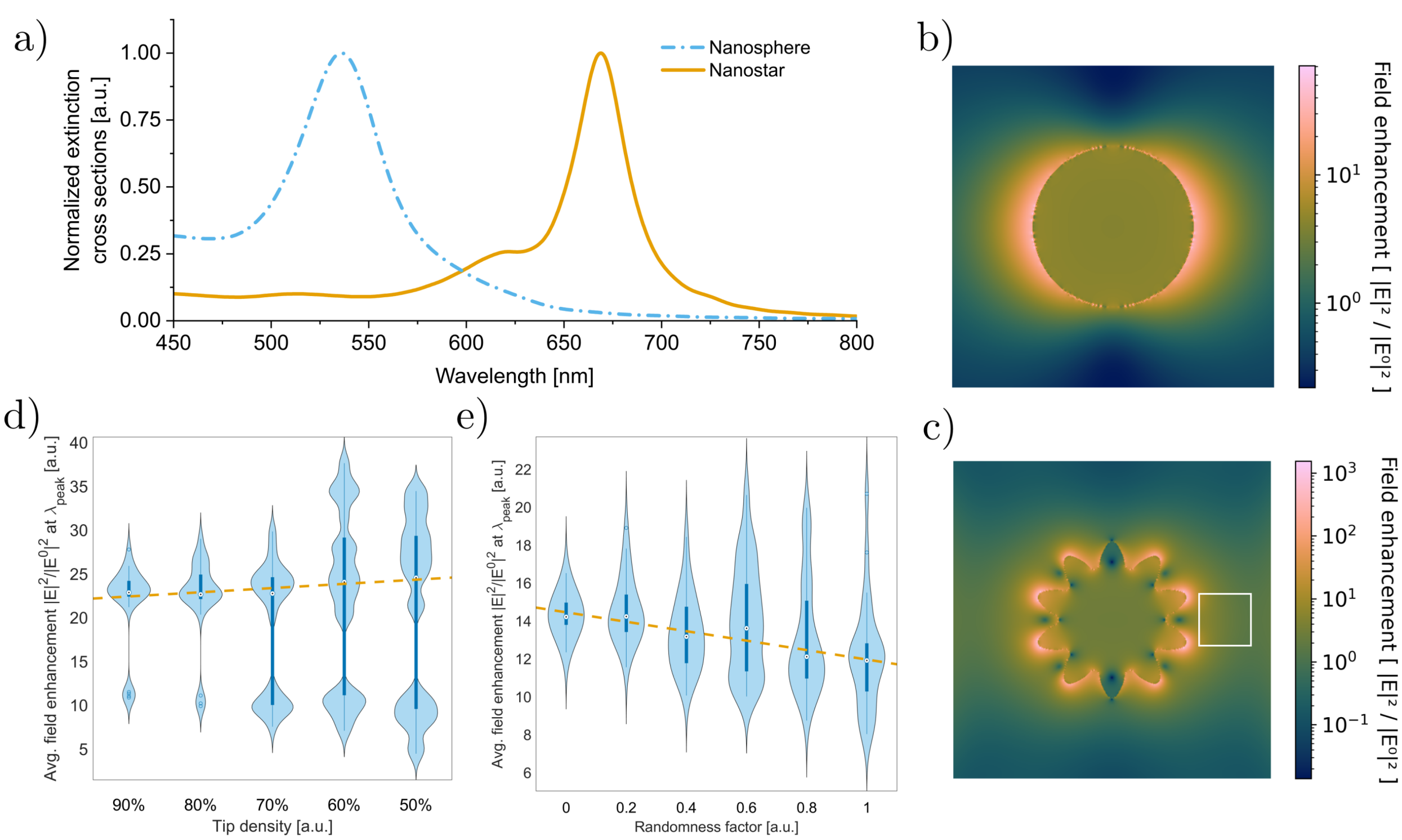

Figure S1: Gold nanostars are optimal candidates for in vivo plasmonic enhancement. a, FDTD simulated extinction cross sections of, respectively, a gold nanosphere and a gold nanostar in a watery environment. The simulated nanosphere has a radius of $25nm$. The simulated nanostar has a core radius of $25nm$ and a tip length of $10nm$. **b,** FDTD simulation of the field enhancement around a gold nanosphere in water at $\lambda = 540nm$. **c,** FDTD simulation of the field enhancement around a gold nanostar in water at $\lambda = 650nm$. The field is confined to a few nanometers around the nanostar spikes. The white square indicates the volume in which the average field enhancement is measured, as explained in the Methods. **d,** FDTD simulations of gold nanostars with a core radius of $30nm$ and a tip length of $10nm$, with different tip densities. For each simulation, the positions of the missing tips and the orientation of the nanostar were randomized. The individual results are shown in the Supplementary Information (**Figure S7**). **e,** FDTD simulations of randomized gold nanostars from an initial ideal particle with a core radius of $30nm$ and a tip length of $10nm$. The tip vertex position and nanostar orientation were randomized for each simulation. The individual results can be found in the Supplementary Information (**Figure S8**).

The starting point for this modelling was an icosahedron shape, with its vertices extended to mimic the tip arrangement found on real nanostars. To ensure smoother surfaces, a Subdivision Surface Modifier was applied. During these simulations, a mesh size of 35 Meshes Per Radius (MPR) was consistently used. For benchmarking, a model nanostar with a core radius R of 25 nm and a tip length L_{tip} of 10 nm was adopted. For the simulations shown in **Figures S1d** and **S1e**, a core radius of 30 nm was used. Simulations explored variations in tip density and randomness to understand their implications for field enhancement. These variations included the random omission of tips and the random displacement of tip vertices. Each group consisted of 25 independent simulations. As far as the random displacement of tips is concerned, for each vertex Blender's "Vertex Randomize" tool sampled a unit-length direction vector uniformly over the sphere and a displacement magnitude m drawn uniformly from the interval $[0, R]$. We normalized R to range from 0 to 1 Blender Distance Units (BDU) and therefore refer to it throughout as the "randomness factor". Since the sharp tips simulated in **Figures S1a**, **S1c**, and **S1d** generated unphysical fields when randomized in Blender, we introduced an adaptation to their sharpness to prevent unrealistic stretching and overly sharp edges. Simulations of gold nanostars in water involved the calculation of the average field enhancement $|E|^2/|E^0|^2$ within a small cubic volume ($\Delta x = \Delta y = \Delta z = R$) located 4 nm from the surface. All scripts for Lumerical and Blender can be found on GitHub.[2]

Spherical gold nanoparticles

Numerical simulations of gold nanospheres were conducted using the script for Lumerical's FDTD Solutions, which is publicly accessible in the GitHub repository.[2]

The code performs simulations by varying the radius R of a sphere submerged in water. It identifies the maximum field enhancement $|E|^2/|E^0|^2$, averaged within a small volume ($\Delta x = R/2$, $\Delta y = R/2$, $\Delta z = R/2$) located 4 nm away from the surface. The resonance wavelength and the corresponding average field enhancement are then recorded in a text file.

This computational "screening" process continues within a specified range, with steps of 2 nm, ranging from $R_{\min} = 6$ nm to $R_{\max} = 76$ nm.

Figure S2 illustrates the relationship between the radius of the sphere and the peak wavelength in the average field enhancement. The simulation results align well with the theoretical model of the Modified Long-Wavelength Approximation (MLWA),[3] indicating the limited tunability of the plasmonic resonance for this simple morphology. This red-shift can be attributed to dynamic depolarization of the nanoparticle.[3]

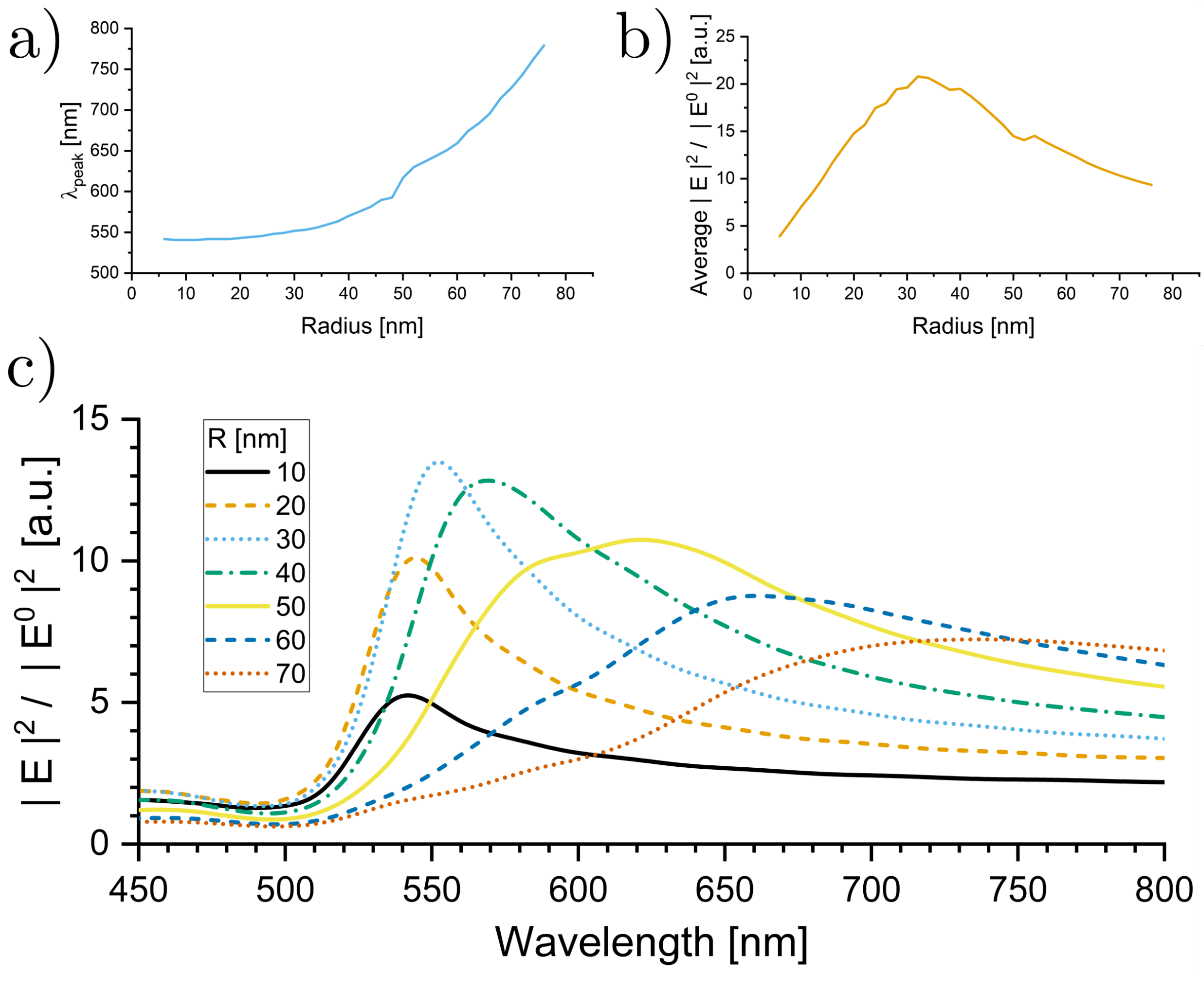

Figure S2: a, Peak position and **b,** intensity of the average field enhancement of gold nanospheres in water, measured in a volume 4 nm away from the surface of the particle, as a function of radius. Larger radii correspond to stronger peak's redshift and lower field enhancement. **c,** Spectra of gold nanospheres immersed in water for different radii. The smaller nanoparticles (10-40 nm) have a resonance peak around 550 nm. The resonance peaks are redshifted for the larger particles, with some even going into the IR region. For particles larger than ~ 30 nm, the width of the peak is significantly broadened.

The dampening and broadening of the field enhancement for particles with radii larger than 30 nm can be attributed to the radiative damping arising from the spontaneous emission of radiation by the induced dipole.[3] These simulations are valuable for verifying the reliability of the employed method and directly computing the local electric field instead of relying on indirect estimates.

The initial findings have brought to light the importance of finding a shape that offers a more tunable and intense plasmonic resonance. This realization has led us to explore the potential of utilizing the peak splitting observed in ellipsoids. The idea is then to identify a shape similar to the ellipsoid, but also practical and straightforward to fabricate with techniques like electron beam lithography.

Smooth gold nanorods

In this section, we focus on realistic nanorods that do not have very sharp edges. In a sense, they are similar to ellipsoids, but present flatter surfaces and have overall larger volumes. These nanorods are particularly interesting due to their unique plasmonic properties and tunability, and could be nanofabricated in regular patterns.

Numerical simulations of gold smoothed nanorods were performed using the script for Lumerical's FDTD Solutions, which is publicly available in the GitHub repository.[2]

In brief, the code varies the three main parameters (namely length l, width w and height H) of the smoothed rod immersed in water and computes the maximum average field enhancement $|E|^2/|E^0|^2$ in a small volume ($\Delta x = w/2$,$\Delta y = w/2$,$\Delta z = H$) 4 nm away from the surface. Then, the resonance wavelength and the corresponding average field enhancement are saved in a text file. This computational screening continues in a defined range by steps of 5 nm:

- 20 nm $\leq l \leq$ 100 nm;
- 20 nm $\leq w \leq$ 50 nm;

- 20 nm $\leq H \leq$ 50 nm;

To save computational time, the script skips tests with higher l if the resonant wavelength is longer than 780 nm (outside the region of interest).

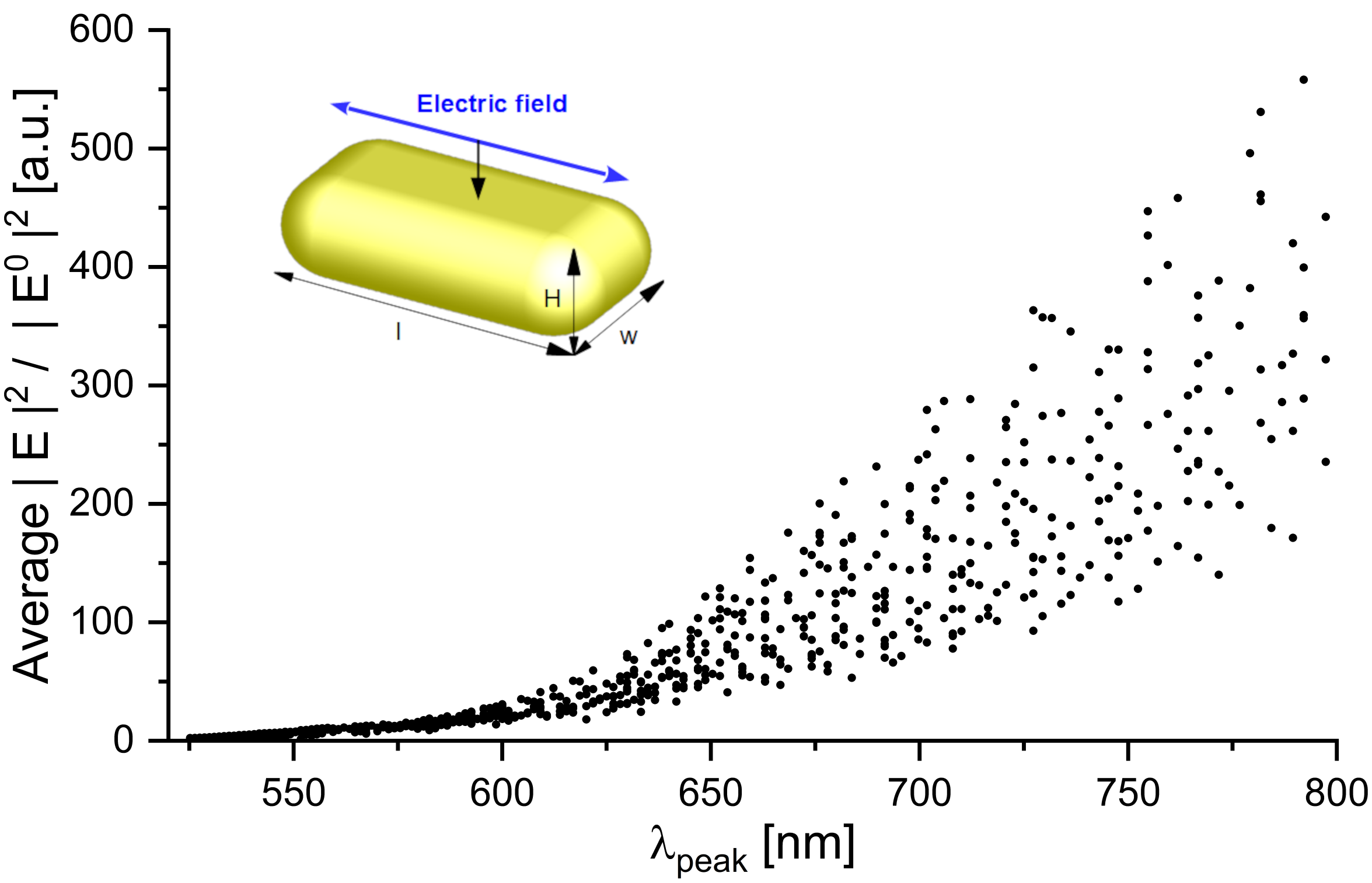

Figure S3: Results of the computational screening of gold nanorods, where each spot corresponds to a set (l,w,H).

In **Figure S3**, each data point corresponds to the field enhancement of an individual nanorod with a fixed combination of dimensions (l, w, H). The ranges chosen for the simulations focused solely on the longitudinal resonance, resulting in the absence of the transverse resonance in the graph. As anticipated by the theory of elongated particles[3] and consistent with previous nanorod experiments,[4] the field enhancement becomes stronger as the resonance is red-shifted. This behaviour is directly related to the aspect ratio of the nanoparticle.

Notably, these simulations deviate from other studies by taking into account various values for the height H rather than treating it as negligible. The effect of height is qualitatively

similar to that of changing the width, contributing to multiple combinations of parameters yielding similar resonance wavelengths.

While the smooth gold nanorods exhibit tunability within a desirable range (near-IR), two main challenges arise with this morphology. First, when these nanorods are nanofabricated on a substrate, the field enhancement remains confined to that plane. Given that biological applications occur in complex three-dimensional environments, this limitation poses a significant obstacle. That is problematic, because if the nanorods are synthesized in a colloidal solution, achieving precise control over their orientation becomes difficult. Consequently, there is a risk of exciting the blue-shifted transverse mode instead of the more intense longitudinal mode.

To address these limitations and build upon the concept of elongated features, we look to spiked nanoparticles as the next logical step. By combining the elongated aspect with the rotational symmetry of spheres, spiked nanoparticles offer promising possibilities. These structures hold the potential to overcome the challenges faced by smooth nanorods and provide enhanced control over plasmonic resonances in three-dimensional environments.

Octahedral silver nanoparticles

Having established a solid understanding of plasmonic resonance in smooth nanoparticles, we can now explore the realm of nanostars, which offer even more significant local enhancement thanks to their sharp tips. An octahedral nanostar structure was employed to address the issue of rotational dependence in the enhancement. This particle exhibits rotational symmetry in multiple directions, simplifying the simulation process. Instead of varying multiple rotation angles, only one angle needed to be adjusted to capture the complete 360-degree behaviour of the enhancement.

For this section, the material of the nanoparticles was arbitrarily chosen as silver instead of gold. Since our focus is primarily on the impact of shape, the choice of material should have minimal influence on our analysis and treatment.

Numerical simulations of silver octahedral nanostars were performed using the script for Lumerical's FDTD Solutions, which is publicly available in the GitHub repository.[2] The conical base was set to have a conical angle of either 25 degrees or 40 degrees. To create the rounded tips, the truncated cone was connected to a semisphere, with the semisphere's radius set to 0.05 times the particle's radius ($0.05R$). Additionally, the length of the spikes was determined to be 1.5 times the particle's radius ($1.5R$). This conical structure more realistically represents a physical nanostar compared to a cone with an infinitely sharp tip.

In order to assess the bandwidth of the enhancement, simulations were conducted by rotating the particle between 0 and 45 degrees. Further rotations beyond this range were deemed redundant due to the 4-fold symmetry of the particle. Throughout the simulations, the monitor remained fixed to maintain consistency. This comparative analysis aimed to provide insights into the distinctive properties and performance of the nanostar structure as a function of rotation.

As visible in **Figure S4a**, there is a strong dependence of the field enhancement intensity on the rotation of the particle. However, the wavelength at which the plasmonic resonance occurs is very stable. Here, the centre spherical particle had a radius of 20 nm, and the spikes had a length of 30 nm; therefore, the characteristic length of the nanostar is $2 \cdot (20+30) = 100$ nm. Therefore, we compare it to a spherical nanoparticle with a diameter of 100 nm to get a rough comparison for the practicality of the nanostar. We want the average enhancement of the nanostar to be higher than the spherical silver nanoparticle.

By calculating the average enhancement per degree over the entire range of rotation, we find an average enhancement of 12 per degree. Since the enhancement of the spherical particle does not change under rotation, the average enhancement is simply the maximum enhancement, so 13.5 per degree. We observe that, on average, the enhancement of the nanostar is less high than the spherical particle in the visible spectrum. In an effort to increase this bandwidth, we tested a different conical base angle of the spikes whilst keeping the radius and spike length the same (**Figure S4c**).

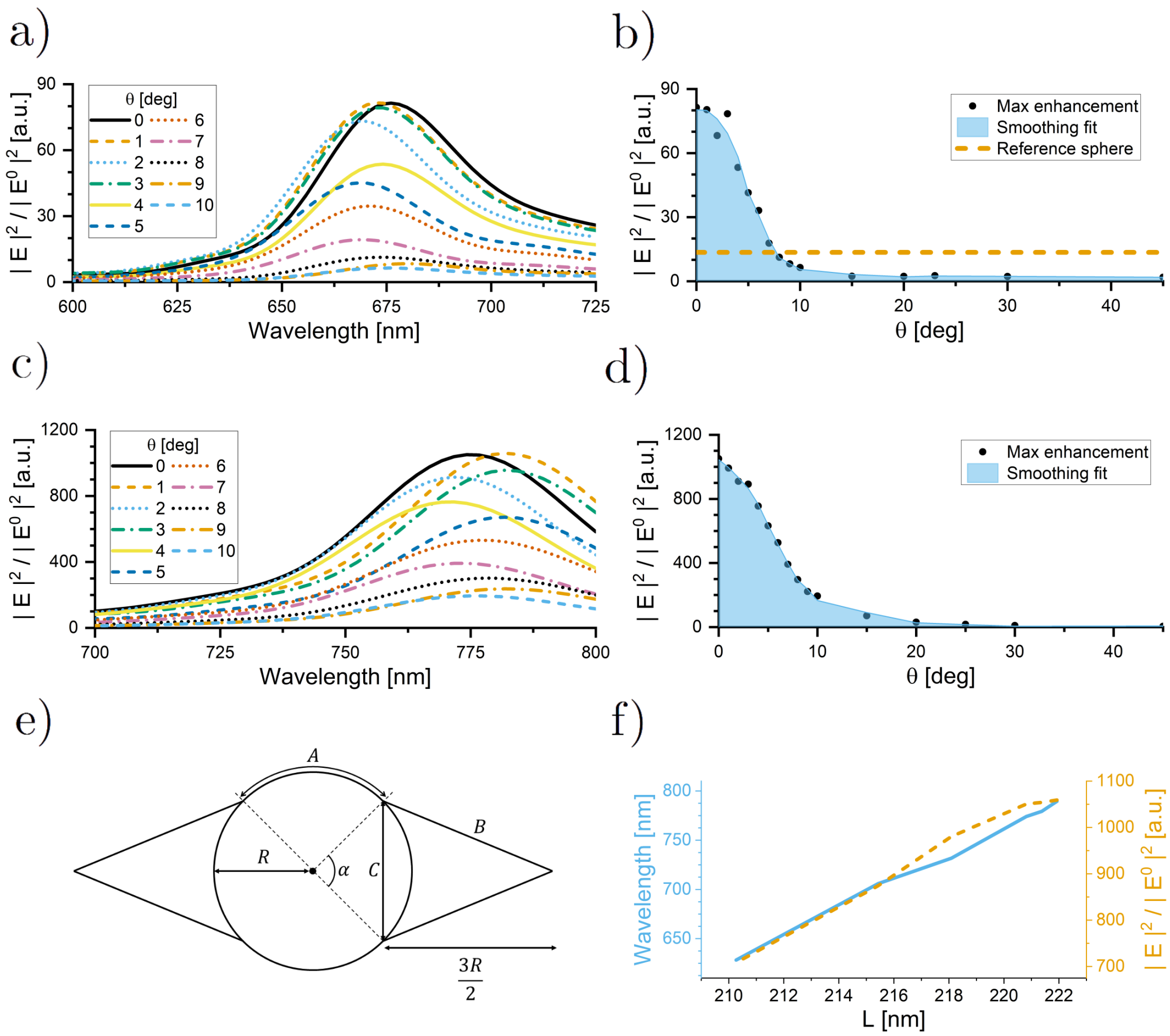

Figure S4: Angles don't significatively change the peak position of the plasmonic resonance, but they affect the local field on-axis. Larger base angles result in red-shifted and more intense plasmonic peaks. Both peak position and field enhancement are linearly dependent on the circumference in the observed plane.

The results reveal a remarkable enhancement peak in the nanostar structure, surpassing previous simulation findings by over an order of magnitude. Additionally, similar to the earlier observations, the wavelength corresponding to this higher enhancement peak remains relatively stable even when the particle is rotated. Regrettably, the spectrum bandwidth remains essentially unchanged compared to the previous simulations. However, the significant increase in enhancement values leads to a noteworthy rise in average enhancement per degree, reaching an impressive value of 167 per degree. Compared to its spherical counterpart with a similar characteristic length (as discussed earlier), the nanostar exhibits a substantially higher average enhancement.

The characteristic length so calculated is independent of the conical aperture of the spikes, therefore not explaining the different behaviours in **Figures S4a** and **S4c**. A different way to evaluate the characteristic length is calculated according to the definitions in **Figure S4e** as the circumference (in the section plane) of the particle. The perpendicular spikes are neglected as they do not affect the plasmonic enhancement (transverse mode).

The formula used to calculate the characteristic length, with α in radians, is:

$$L = 2R\left[\pi - \alpha + 2\sqrt{\sin^2\left(\frac{\alpha}{2}\right) + \frac{9}{4}}\right] \tag{S.1}$$

We considered conical base angles ranging from 38 to 60 degrees, as these angles correspond to peaks within the visible spectrum. Using **Equation (S.1)**, we calculate the circumference lengths and plot them against both the resonance wavelength and the maximum enhancement in **Figure S4f**. Both the maximum enhancement and the peak resonance wavelength exhibit a linear relationship with the circumference length.

Figure S5 clearly demonstrates the persistence of an intense plasmonic effect in the spiked nanoparticle; however, the localization of this effect at the tip implies that the measured field within a fixed window will significantly decrease as the nanoparticle undergoes rotation.

Given the orientation-dependent nature of octahedral nanoparticles, we contemplated the idea of utilizing a spiked nanoparticle with a high density of tips as a potential solution to overcome this challenge. The abundant spikes in such a structure could offer advantages in terms of orientation insensitivity, allowing us to explore plasmonic properties more effectively.

Highly spiked gold nanostars

Numerical simulations of highly spiked gold nanostars were conducted using the script for Lumerical's FDTD Solutions, which is publicly available in the GitHub repository.[2]

In brief, the code performs simulations by varying the core radius R of the nanostar

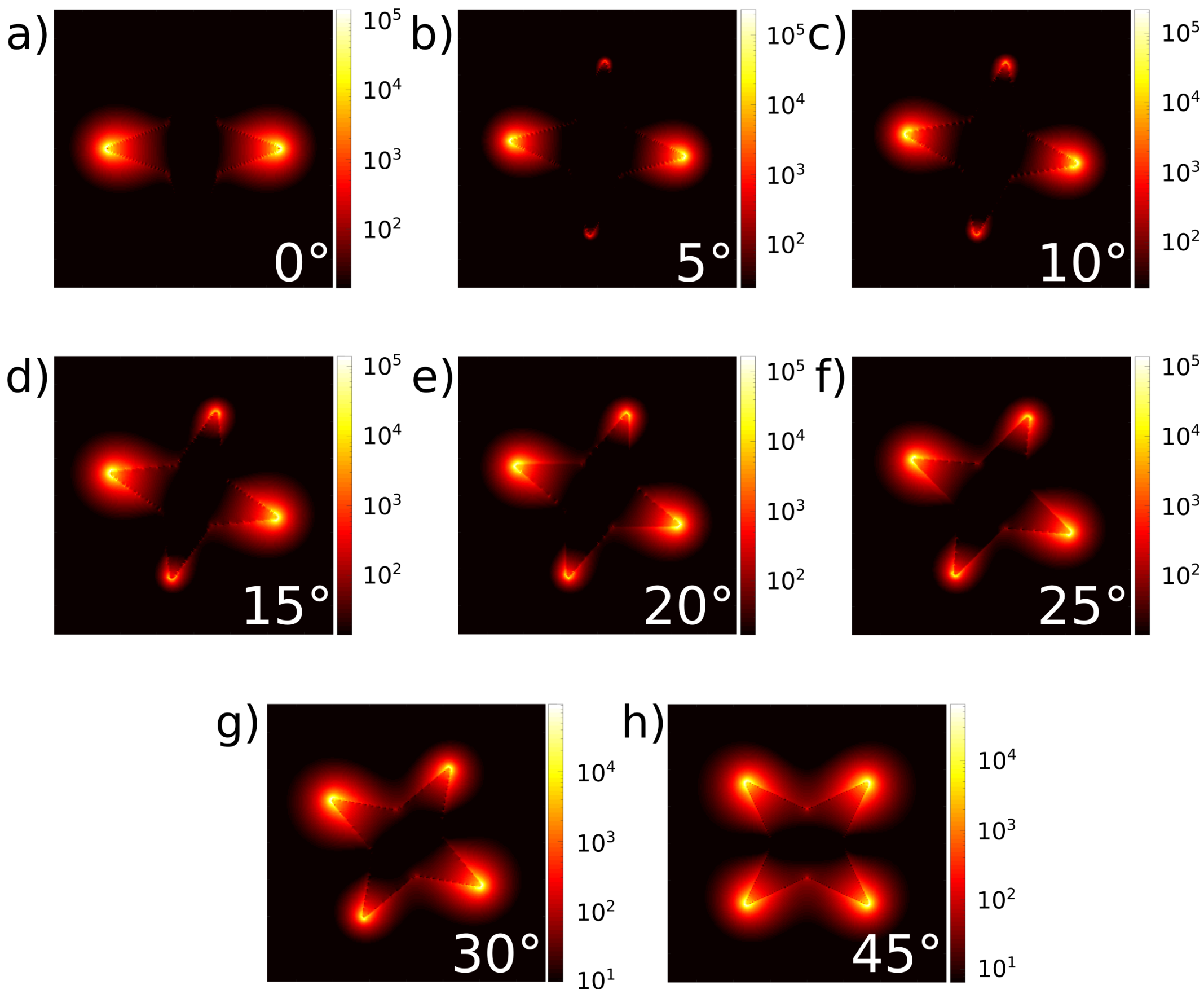

Figure S5: Field enhancement maps for a silver octahedral nanostar with 40 degrees of conical angle, for different rotations, at the resonance wavelength (774 nm). The maximum enhancement close to the tip is roughly the same order of magnitude, but this localization results in a lower field enhancement on the x-axis for some rotation angles.

immersed in water, keeping the tip length fixed at 10 nm. The primary goal is to compute the average field enhancement $|E|^2/|E^0|^2$ within a small volume ($\Delta x = R_{\text{core}}, \Delta y = R_{\text{core}}, \Delta z = R_{\text{core}}$) positioned 4 nm away from the surface. Subsequently, the resonance wavelength and the corresponding average field enhancement values are recorded in a text file. Additionally, the code saves the absorption and scattering cross-sections as functions of the wavelength. The simulations cover a defined range, incrementing the core radius by 5 nm, spanning from a minimum core radius of $R_{\text{core,min}} = 20$ nm to a maximum core radius of $R_{\text{core,max}} = 50$ nm.

The presence of the tips in the nanostars, as anticipated from the theory,[5] leads to an immediate red shift of approximately 100 nm compared to the plasmonic resonance observed in gold spherical particles of the same size. Unlike nanospheres, nanostars exhibit a more

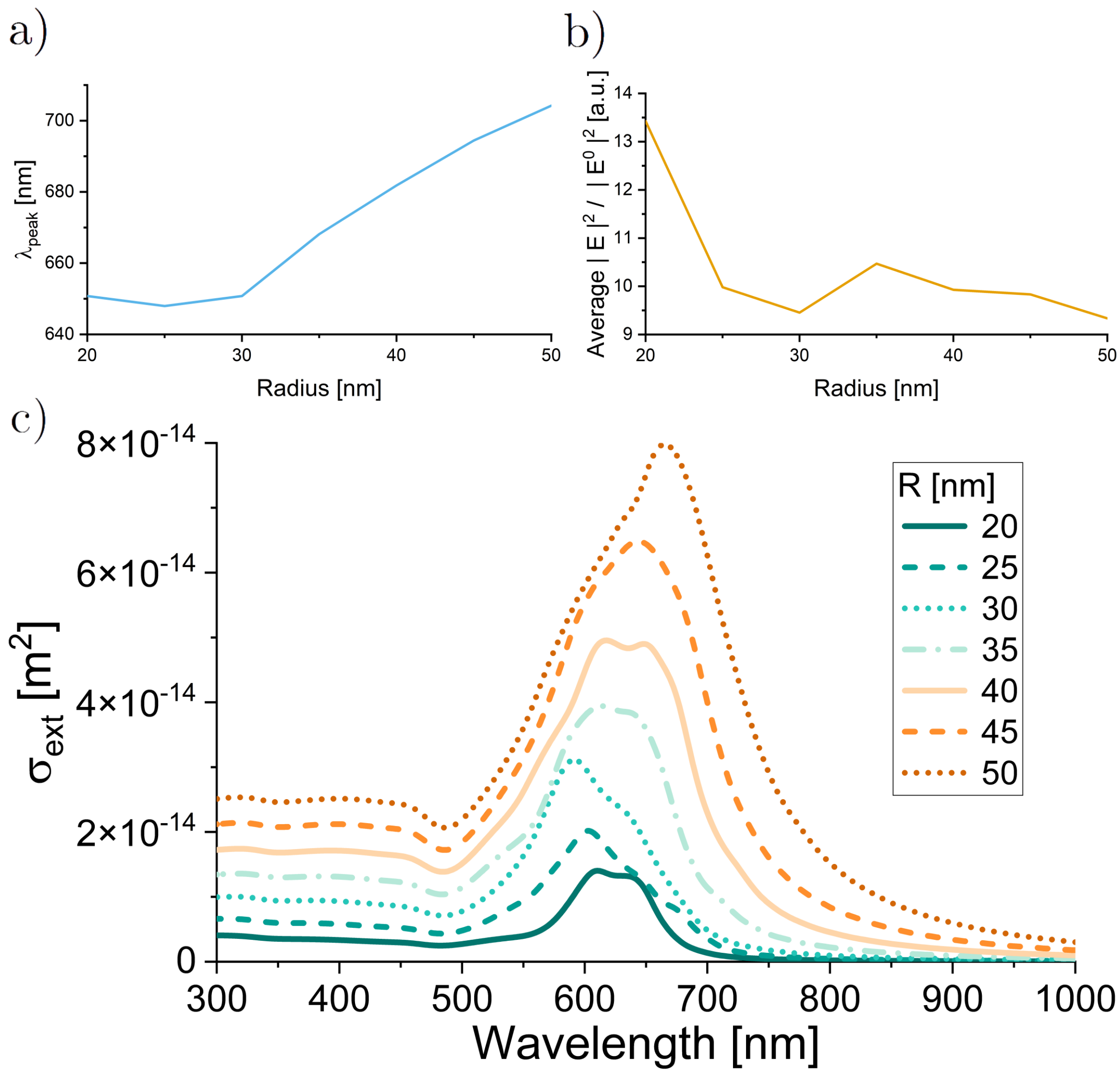

Figure S6: Screening of gold nanostars (high spike density) in water; larger radii correspond to stronger peak's redshift. Field enhancement is not significantly dependent on core size.

limited shift in resonance wavelength when the core size is altered, allowing for finer tunability of the peak. The initial blue shift is attributed to the competing effects of absorption and scattering, as illustrated in **Figure S6**.

Interestingly, due to the presence of the spikes, the absorption peak consistently appears blueshifted relative to the scattering peak. Consequently, this results in a general blue shift when larger particles are grown, followed by a subsequent red shift. **Figure S6** offers a comprehensive comparison, outlining the primary effects of growing larger nanostars. The most notable effect is the significant broadening of the peak, resulting from non-dipolar

effects, which leads to a less selective plasmonic resonance.

The simulation work focusing on highly spiked nanostars, characterized by a high density of tips around the spherical core, primarily remained theoretical. Nevertheless, these simulations already indicated the potential of such a morphology in achieving a favourable combination of resonance control, field enhancement engineering, and insensitivity to rotations. To make the study more relevant and applicable to real-world scenarios, it became essential to identify an existing synthesis protocol capable of producing gold nanostars with the plasmonic peak positioned in the red region of the visible range. Thus, to bridge the gap between theory and practicality, we adapted and applied these simulations to realistic gold nanostars as discussed in the main manuscript.

Individual simulations of realistic nanostars

Here reported are the individual simulation results, which were then aggregated in **Figures S1d** and **S1e**.

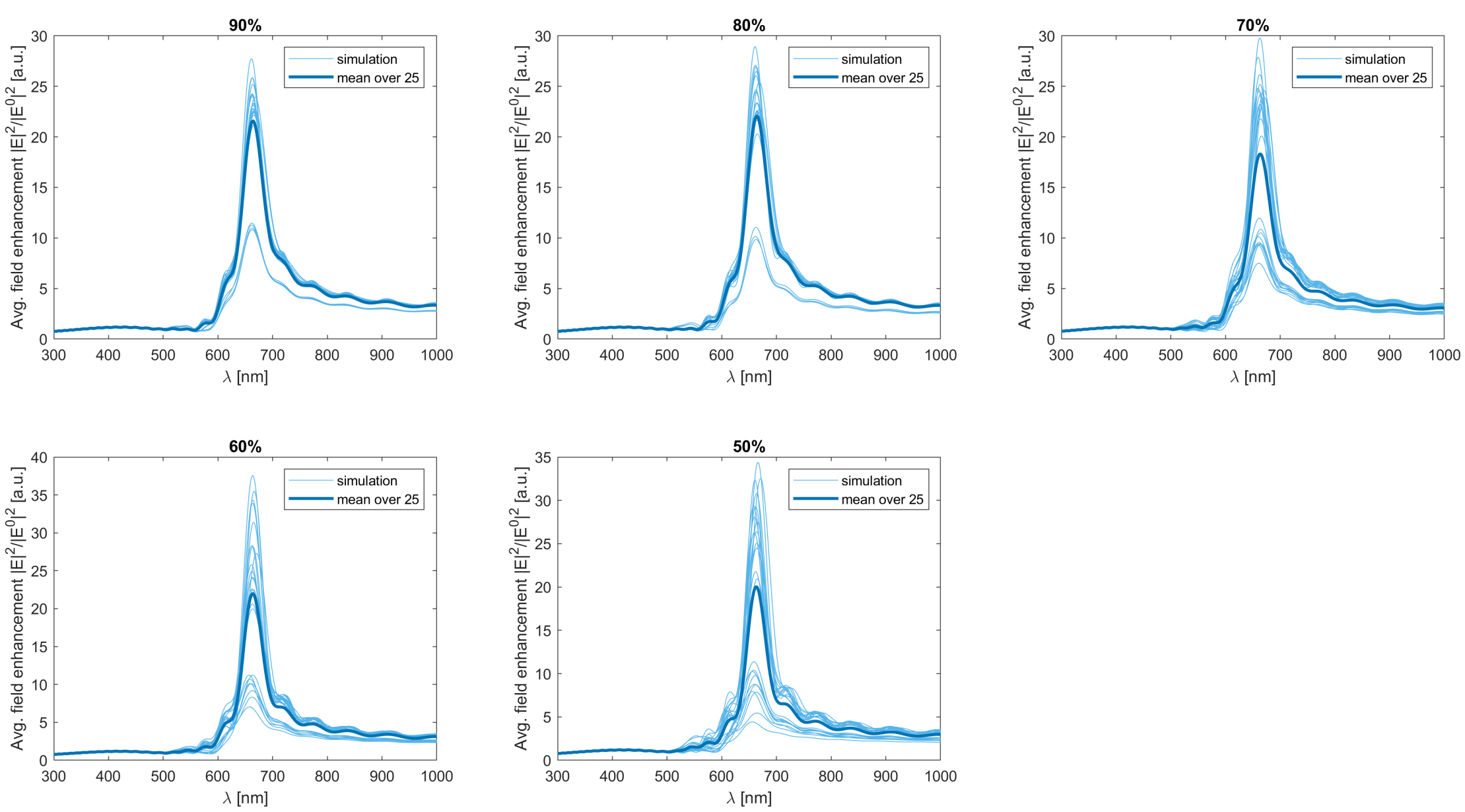

Figure S7: Invididual simulation results at different tip lengths. The results are aggregated in **Figure S1d**.

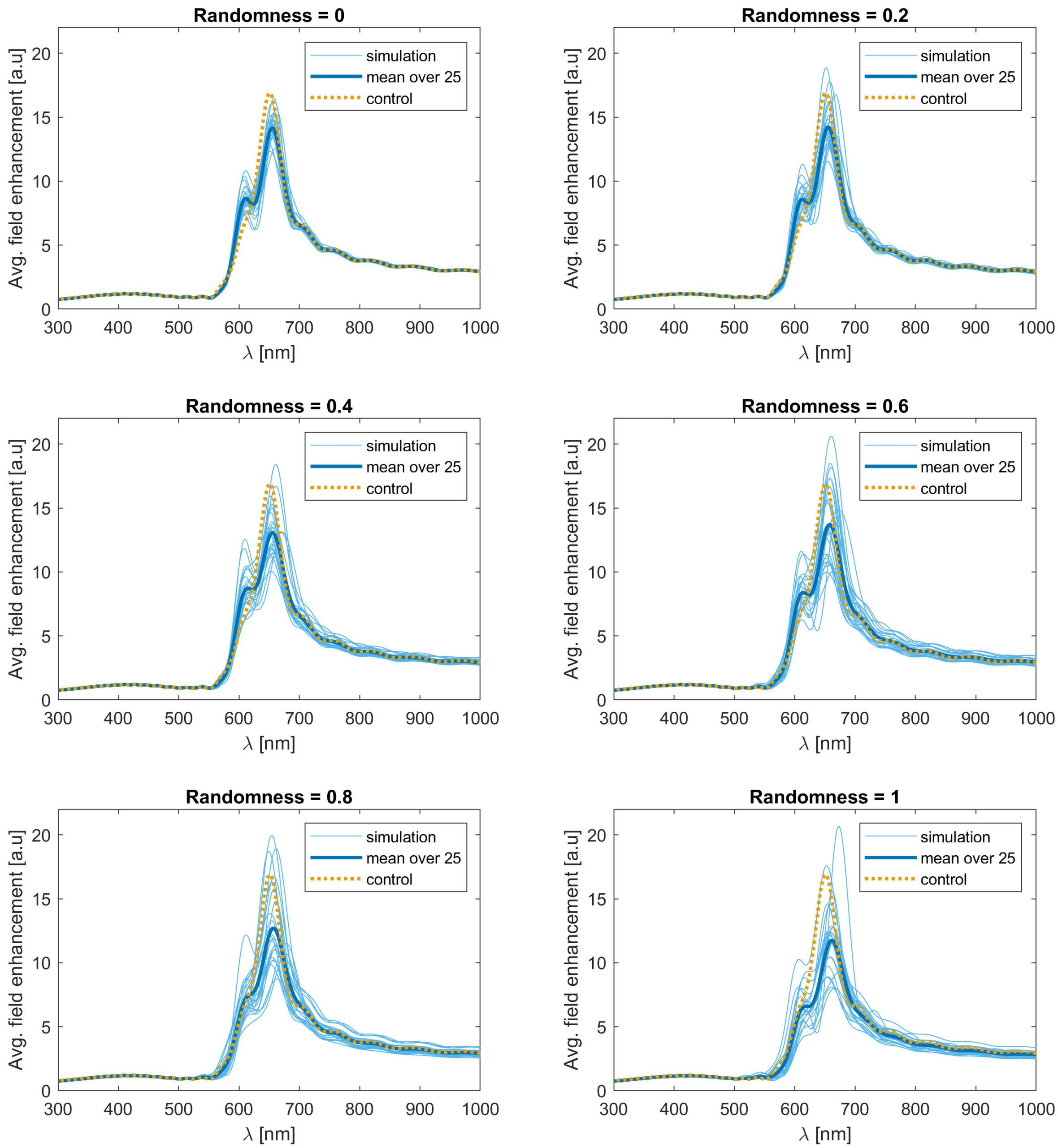

Figure S8: Invididual simulation results at different randomness factors. The results are aggregated in **Figure S1e**. The control corresponds to an ideal nanostar, optimally aligned with the field monitor.

Synthesized gold nanostars

Colloidal synthesis

Gold is a common metal for plasmonic applications, characterized by superior biocompatibility and low cytotoxicity,[6] and was therefore the material of choice used throughout this study. We synthesized a series of nanostars under varying seed and growth conditions that allowed high control and tunability of the geometry. In the presence of a high concentration of polyvinylpyrrolidone (PVP) dissolved in dimethylformamide (DMF), highly branched nanostars can be obtained efficiently at room temperature by optimizing the molar ratio R=[$HAuCl_4$]/[seeds].[7] Gold tips can grow on pre-formed gold seeds thanks to the reducing power of DMF and the excess of PVP. The protocol for making PVP-coated gold nanostars was adapted from the seed-mediated synthesis proposed by Barbosa et al.[8] For each trial, PVP solubilized in DMF (15 mL, 10 mM; Sigma-Aldrich, PVP10, D4551) was transferred into a 25 mL conical flask under continuous magnetic stirring. Then, an acqueous solution of hydrogen tetrachloroaurate(III) ($HAuCl_4$; 82 µL, 50 mM; Sigma-Aldrich, 520918) was added. Within 30 s, a predetermined volume of gold spherical seeds (15 nm diameter; 0.5 mM; in-house synthesis based on Barbosa et al.[8]) was added. The volume of pre-formed seeds was determined based on the formula $V_{\text{seed}} = \frac{[\text{HAuCl}_4]\cdot V_{\text{HAuCl}_4}}{[\textbf{seeds}]\cdot R} = \frac{8.2\text{mL}}{R}$, where R is the desired molar ratio. After 15 minutes under continuous stirring, the samples were spun at 4000 rpm for 90 minutes and the supernatant was removed to prevent the undesired formation of suboptimal nanoparticles. The nanostars were re-dispersed in MilliQ water (15 mL, final concentration 5.3e10 particles/mL) and vortexed for a couple of seconds. This last purification step was carried out twice to minimize the cytotoxic effect of DMF and to reduce the amount of PVP in the solution. The optimal sample used throughout the manuscript corresponds to a molar ratio of 30 (273 μL of seeds).

Energy-dispersive X-ray spectroscopy of gold nanostar colloids

We characterized the synthesized colloids through Energy Dispersive X-ray Spectroscopy (EDX, **Figure S9**). This substantiated the purity of the gold nanostars, with no residual gold detected in the sample (**Figure S9**). EDX measurements were performed in a Thermo Fisher Scientific (TFS) Nova 600i Nano Lab dual-beam system using an Oxford Instruments X-MAX 80 EDX detector. The working distance was kept at 5 mm to have the optimum EDX signal. Spectra were analyzed using the Oxford Instruments AZtec software. The reported map EDX was obtained with a primary beam energy of 5 kV. Si and Au are mapped and reported in weight percentage (Wt%).

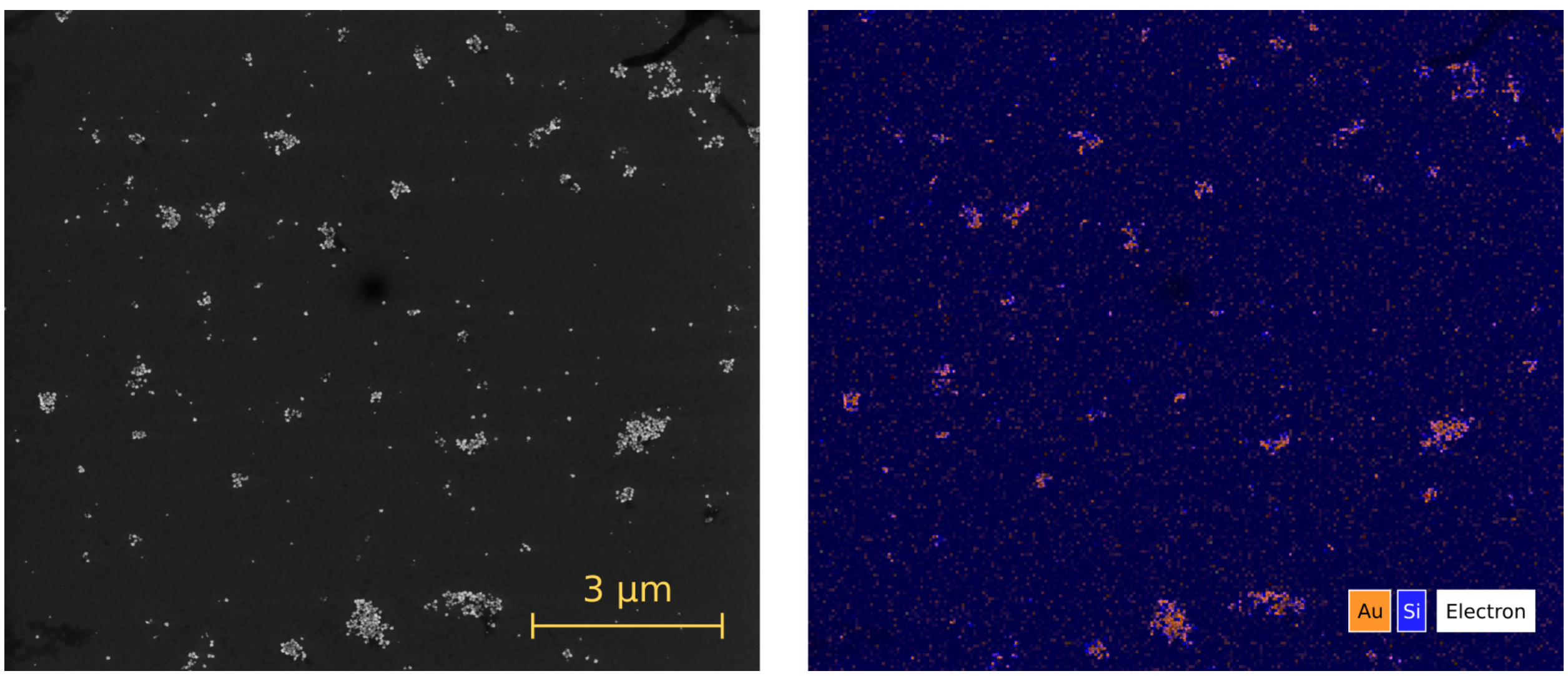

Figure S9: Validation of the purity of gold nanostar samples, showing the absence of any residual gold in the specimen. On the left, the SEM micrograph of a nanostar sample bound to glass via MPTMS; on the right, the same field of view under EDX inspection.

UV/Visible spectroscopy of gold nanostar colloids

We characterized the synthesized colloids through Ultraviolet-Visible light (UV-Vis) spectroscopy. The optimal sample (molar ratio $R = 30$) was selected based on the alignment of the absorbance peak to the emission peak of Archon1[9] (**Figure S10a**). We attribute a minor mismatch to a red shift induced by polyvinylpyrrolidone (PVP) as a capping agent

(**Equation (S.4)**) The absorbance spectra were measured using a compact Visible-NIR light source (ecoVIS by Ocean Optics) together with a compact STS spectrometer (STS-VIS Microspectrometer, 350 – 800 nm, by Ocean Insight). Absorbance was measured using an integration time of 5.1 ms after background subtraction and intensity calibration, and averaged 1000 times. Absorbance spectra of 50 nm and 90 nm off-resonant nanoparticles (**Figure 3h**) were measured using a quartz tungsten-halogen light source (SLS201L/M, ThorLabs), connected to a cage cube (CVH100/M, ThorLabs) via a 400 µm Visible-NIR fiber (P400-1-VIS-NIR, Ocean Optics), which was in turn connected to a 200-1000 nm compact spectrometer (CCS200/M, ThorLabs) via a 1500 µm 0.39 NA SMA fiber patch cable (M39L01, ThorLabs). 400 µL of nanoparticle suspension or reference solution was added to a 1.5 mL Poly(methyl methacrylate) cuvette (759085D, BrandTech), which was placed in the cage cube for measurement. As reference solution, phosphate buffered saline (20012-019, Gibco) was used. Absorbance was measured using an integration time of 1.8 ms and averaged 300 times.

SEM characterization of gold nanostar colloids

We performed quality control by immobilizing the gold nanostars on a silicon substrate with MPTMS (**Figure S10b**), following the protocol outlined in the main manuscript, to examine them under a scanning electron microscope (SEM). The monodisperse nanostars had an average core radius of $r = 23 \pm 5$ nm (mean ± SD, **Figure S10c**) and an average tip length $l = 12 \pm 3$ nm, (mean ± SD, **Figure S10c**) in agreement with the simulations. They were generally well separated (**Figure S10d**), though occasional clustering in micrometer-sized aggregates was visible (**Figure S10e**), possibly affecting the measured plasmonic resonance.[10] SEM imaging was performed in a Thermo Fisher Scientific (TFS) Nova 600i Nano Lab dual-beam system with a primary beam energy of 5 kV and nominal current of 0.27 nA using the SE-TLD detector. All imaging was performed at 5mm working distance and in immersion mode. Horizontal field of view and dwell time were varied and are specified on

each reported image.

For analysis of the SEM images, We aimed to isolate individual nanoparticles in SEM images to characterize them based on their core radius and tip length. For that purpose, an algorithm was created using Python and OpenCV, available on GitHub.[2] Firstly, the algorithm segmented the nanoparticles from the background using an Otsu adaptative threshold for bi-multimodal histograms. Secondly, an erosion was executed on the binary mask with 5 iterations and a 3×3 kernel to separate close nanoparticles. This way, we isolated the center of mass of the nanoparticles to iterate over the individual contours and generate individual masks for each nanoparticle; on the individual mask, we performed a symmetric dilatation to revert the previous erosion. Next, from the mask of the individual nanoparticles, we calculated the inner maximum radius to find the core size and the minimum enclosing circle to get the total radius and the tip length. Finally, we extracted the pixel/meters conversion factor using the images' metadata for further analysis.

Aggregates that were still detected as single nanoparticles have been filtered post-analysis by applying the 2σ criterion on both radius and tip length.

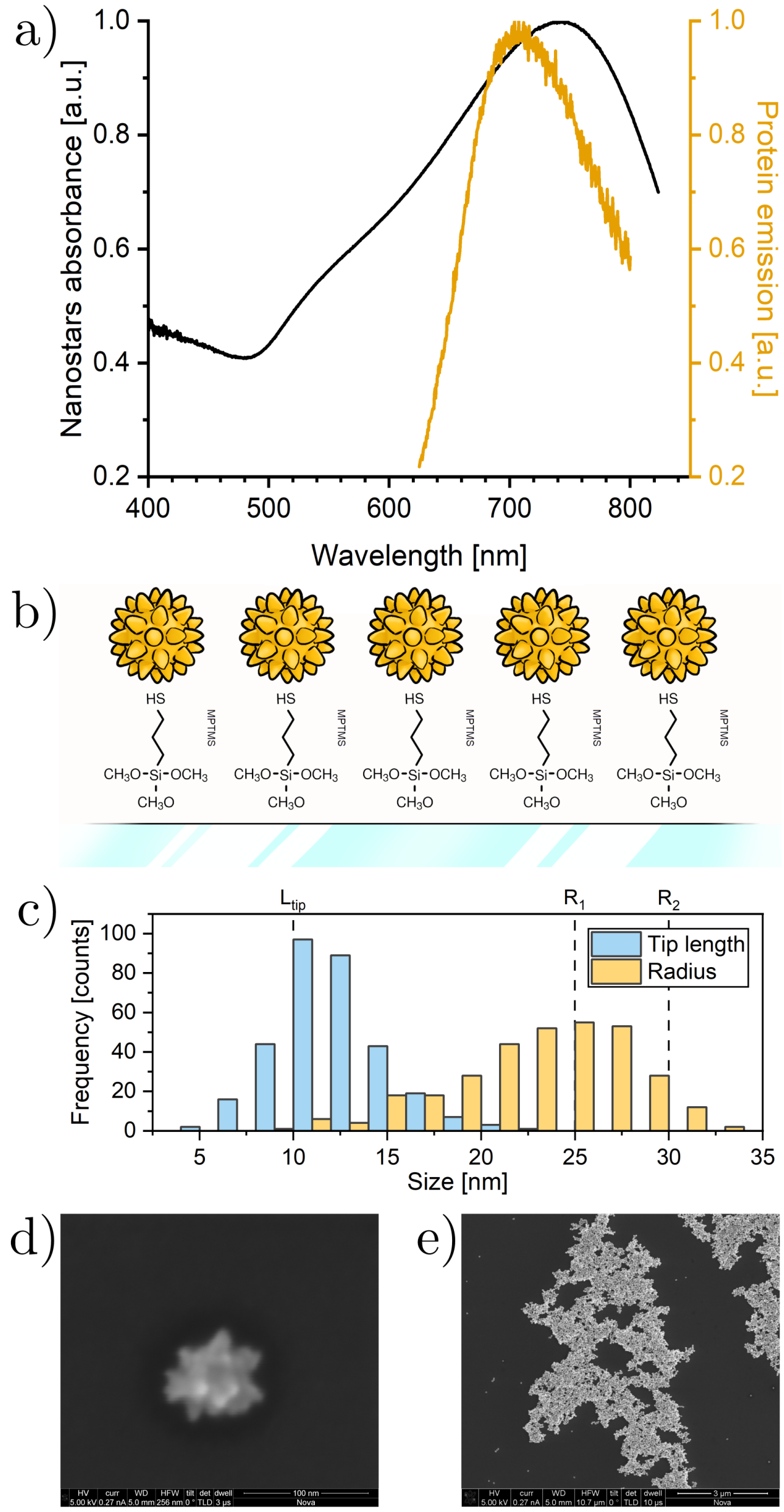

Figure S10: Colloidal gold nanostars are designed to enhance the fluorescence of QuasArs. **a,** Spectral alignment between the normalized absorbance of the gold nanostars colloid and the fluorescence emission of Archon1.[9] **b,** Illustration of the gold nanostars bound to glass via MPTMS. Not in scale. **c,** The physical features of the nanoparticles in SEM images match the ones used for the simulations in **Figure S1**. The average core radius results $r = 23 \pm 5$ nm (mean $\pm$ SD) and the average tip length $l = 12 \pm 3$ nm, (mean $\pm$ SD). In the simulation, $L_{tip} = 10$ nm is the tip length and $R_1 = 25$ nm and $R_2 = 30$ nm are the two radii used in different simulations. **d,** SEM micrograph of an isolated gold nanostar. **e,** SEM micrograph reveals occasional clustering of gold nanostars in micrometer-sized aggregates.

Fröhlich-like shift due to PVP coating

The local dielectric environment has an effect on the plasmonic resonance frequency. To estimate this in the case of PVP-capped nanoparticles, let us assume that the red-shift due to the capping agent is Fröhlich-like:[3]

$$\lambda_{PVP} = \frac{2\pi c\sqrt{\epsilon_{m,\infty} + 2\epsilon_{PVP}}}{\omega_P} \tag{S.2}$$

where λ_{PVP} is the resonance wavelength when considering the PVP as a capping agent, c the speed of light, $\epsilon_{m,\infty}$ the core polarization of gold, ϵ_{PVP} the dielectric constant of PVP and ω_P the plasma frequency of gold.

If the nanostars would be "bare", or simply said immersed in water without any capping agent, the resonance wavelength would be:

$$\lambda_{H_2O} = \frac{2\pi c\sqrt{\epsilon_{m,\infty} + 2\epsilon_{H_2O}}}{\omega_P} \tag{S.3}$$

We can then rewrite λ_{H_2O} in terms of λ_{PVP}:

$$\lambda_{H_2O} = \lambda_{PVP}\frac{\sqrt{\epsilon_{m,\infty} + 2\epsilon_{H_2O}}}{\sqrt{\epsilon_{m,\infty} + 2\epsilon_{PVP}}} \tag{S.4}$$

The plasmonic peak of nanostars coated in PVP is measured experimentally to be at 740 nm. For gold $\epsilon_{m,\infty} = 7.9$,[11] for water $\epsilon_{H_2O} = 1.77$[12] and for PVP $\epsilon_{PVP} = 2.33$.[13]

Plugging these values in **Equation (S.4)** yields $\lambda_{H_2O} = 706$ nm, which aligns almost perfectly with the position of the peak of Archon1[9] and could potentially correspond to the condition where the nanostars are immobilized on the glass substrates.

Estimation of the quantum yield enhancement of Cy5

To estimate quantitatively the fluorescence enhancement of Cy5 when bound to a nanostar via bBSA (**Figure 2c** of the main manuscript), we calculated the average field enhancement

in a ring at 13 nm from the surface to better represent a realistic effect (ring thickness 1 nm, 13 nm away from the surface).

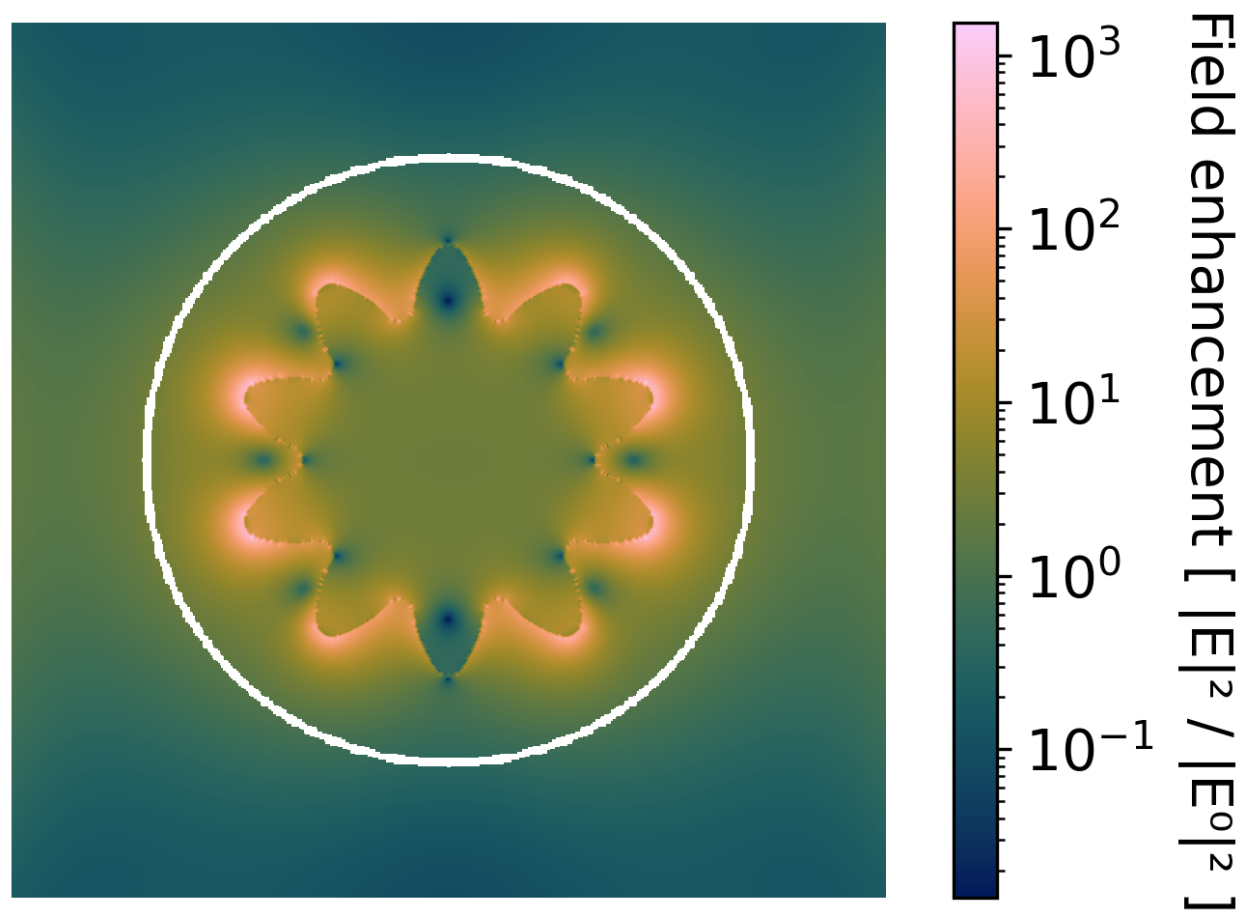

Figure S11: In white, the anular region around the nanostar where the field enhancement is averaged. Ring thickness 1 nm.

The average value in the ring is 1.78 (**Figure S11**). Then, from a reference[14] we took $Y = 0.30$ and $\tau = 2.33$ ns and calculated:

$$\Gamma = \frac{Y}{\tau} = 0.129 \text{ ns}^{-1} \tag{S.5}$$

$$\Gamma_{nr} = \frac{1 - Y}{\tau} = 0.300 \text{ ns}^{-1} \tag{S.6}$$

Given that the main effect is the enhancement of the radiative decay rate Γ, we can calculate the enhanced quantum yield using **Equation (1)** (main manuscript):

$$Y_{enh} = \frac{\frac{|E|^2}{|E^0|^2} \cdot \Gamma}{\frac{|E|^2}{|E^0|^2} \cdot \Gamma + \Gamma_{nr}} = \frac{1.78 \cdot \Gamma}{1.78 \cdot \Gamma + \Gamma_{nr}} = 0.434 \tag{S.7}$$

the relative increase in the quantum yield is 45%, exactly what we measured.

Estimation of the glycocalyx spacing effect

Using the plasmonic ruler as calculated in the main manuscript, we can estimate the spacing due to the glycocalyx by comparing the field enhancement above and below the HEK cells. We use the quantum yield of Q6a ($Y = 0.0367$).[15]

We know that:

$$Y = \frac{\Gamma}{\Gamma + \Gamma_{nr}} \Rightarrow \Gamma = \frac{Y}{1 - Y}\Gamma_{nr} \tag{S.8}$$

and from our experiments:

$$Y_{below} = 1.69Y = 0.062 \tag{S.9}$$

$$Y_{above} = 1.28Y = 0.047 \tag{S.10}$$

The enhanced quantum yield due to field enhancement α is:

$$Y^* = \frac{\alpha\Gamma}{\alpha\Gamma + \Gamma_{nr}} = \frac{\alpha Y}{\alpha Y + (1 - Y)} \tag{S.11}$$

Therefore we can express the field enhancement as:

$$\alpha = \frac{Y^*}{1 - Y^*} \cdot \frac{1 - Y}{Y} \tag{S.12}$$

For the case of nanostars embedded in the fibronectin coating, we get:

$$\alpha_{below} = 1.73 \tag{S.13}$$

which corresponds, according to the plasmonic ruler, to 13.5 nm. For the case of nanostars dropped above the cells, we get:

$$\alpha_{above} = 1.29 \tag{S.14}$$

which corresponds, according to the plasmonic ruler, to 16 nm. Knowing that the fibronectin is 6 nm thick (**Figure 1**), the difference in thickness of the glycocalyx can be estimated to

be:

$$\Delta d = (16 - (13.5 - 6))nm = 8.5nm \quad (S.15)$$

Temperature tests

To determine whether the observed fluorescence enhancement could be attributed to local heating, we employed Rhodamine B, a well-characterized temperature-sensitive fluorophore whose quantum yield decreases linearly as temperature rises.[16] Rhodamine B is relatively insensitive to pH (above 6) and pressure, and its absorption (554 nm) and emission (576 nm) peaks make it suitable for optical thermometry. Owing to its ability to cross biological membranes, Rhodamine B has also been widely used in live-cell applications.[17] Calibration experiments have shown that Rhodamine B fluorescence intensity declines by approximately $1.63 \pm 0.2\,\%$ per °C.[18]

In our control experiment (**Figure S12** and **Figure 3e**), wild-type HEK293T cells without QuasAr6a were plated on glass dishes coated either with gold nanostars by MPTMS or only with MPTMS (no nanostars). Cells were illuminated with a 639 nm laser in alternating 10 s ON / 10 s OFF cycles, and changes in Rhodamine B fluorescence were monitored to detect any heating-related decrease. The fluorescence change in the MPTMS-only control sample was $-11.7 \pm 1.7\%$, while the nanostar sample showed $-14.5 \pm 0.58\%$. Using the reported sensitivity of $1.63 \pm 0.2\,\%$ per °C, these correspond to temperature increases of 7.2 ± 1.4 °C and 8.9 ± 1.1 °C, respectively. The difference between the two groups was not significant(**Figure 3e**), but for the sake of completeness we report that nominally, there was a 2.7% difference in fluorescence between the groups, corresponding to a temperature difference of 1.74 °C. This minimal difference in heating between nanostar and control samples indicates that the presence of nanostars does not produce significant additional local heating under our experimental conditions. We therefore conclude that the fluorescence enhancement observed in the QuasAr experiments cannot be attributed to thermal effects.

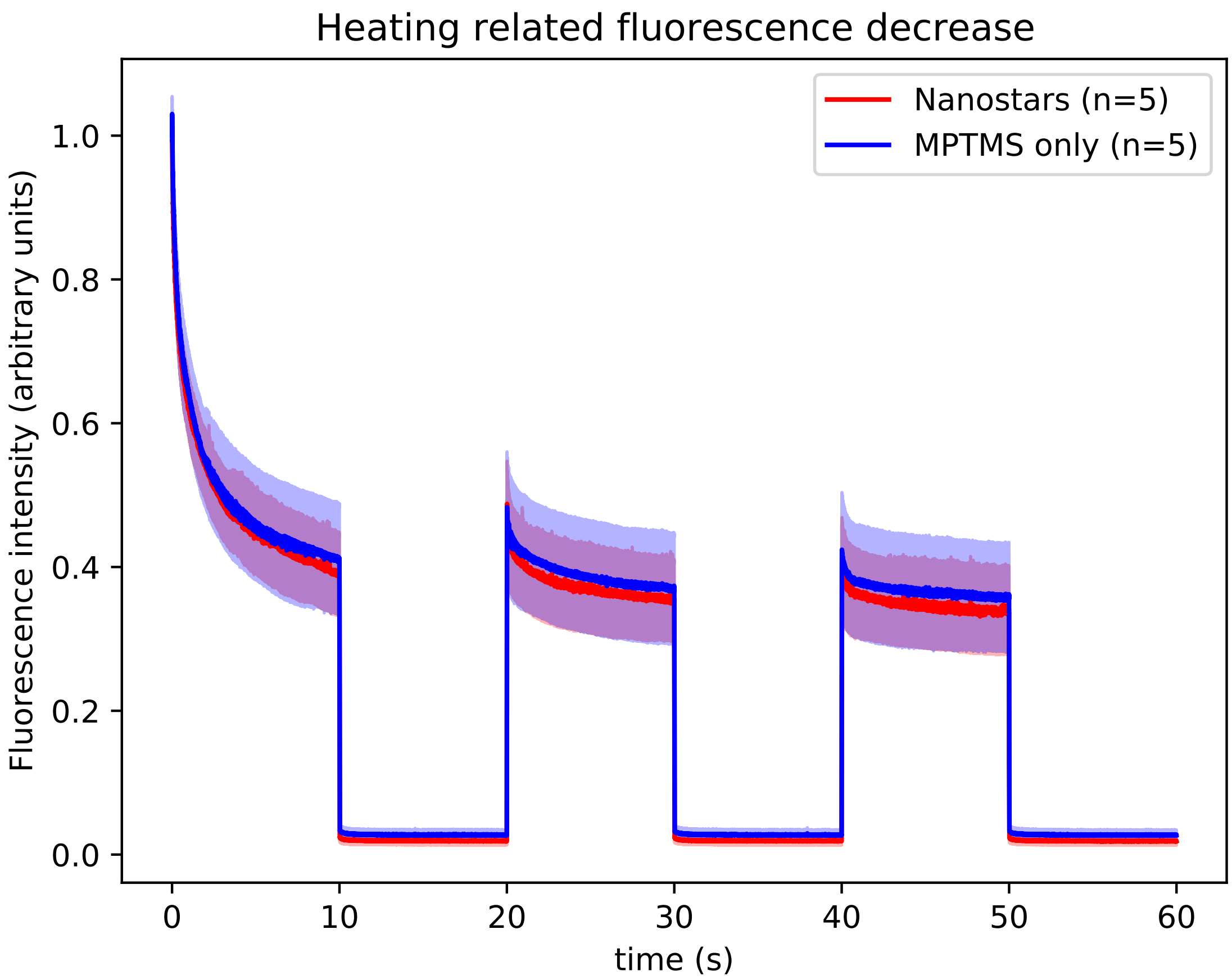

Figure S12: Representative fluorescence traces of Rhodamine B-stained wild-type HEK293T cells plated on dishes either coated with nanostars (red) or MPTMS only (blue). Cells were illuminated with a 639 nm laser in alternating ON/OFF cylces (50% duty cycle, period 20 s). Fluorescence intensities were background-subtracted and normalized to the first frame ($F/F_0 = 1$ at $t = 0$). Shaded areas represent SEM across 5 recordings each.

Photocycle fitting

The quantum yield of the fluorescent state is:

$$Y = \frac{\Gamma}{\Gamma + \Gamma_{nr}} \tag{S.16}$$

The enhanced quantum yield due to field enhancement α is:

$$Y^* = \frac{\alpha\Gamma}{\alpha\Gamma + \Gamma_{nr}} \approx \alpha Y \tag{S.17}$$

The approximation is valid for small Γ/Γ_{nr}. The measured fluorescence is the product of some efficiency factor (system dependent) η, the occupancy of state Q and the quantum yield Y:

$$F = \eta Q Y \tag{S.18}$$

$$F^* = \eta Q^* Y^* = \eta Q^* \alpha Y \tag{S.19}$$

Brightness enhancement:

$$\frac{F_l^* - F_l}{F_l} = \frac{\eta Q_l^* \alpha Y - \eta Q_l Y}{\eta Q_l Y} = \alpha \frac{Q_l^*}{Q_l} - 1 \tag{S.20}$$

Voltage sensitivity (control):

$$\frac{F_h - F_l}{F_l} = \frac{\eta Q_h Y - \eta Q_l Y}{\eta Q_l Y} = \frac{Q_h}{Q_l} - 1 \tag{S.21}$$

Voltage sensitivity (enhanced):

$$\frac{F_h^* - F_l^*}{F_l^*} = \frac{\eta Q_h^* \alpha Y - \eta Q_l^* \alpha Y}{\eta Q_l^* \alpha Y} = \frac{Q_h^*}{Q_l^*} - 1 \tag{S.22}$$

These quantities so defined can be used to directly compare the features between measurements (where F is known, but not the photocycle state population Q) and the simulations (where Q is known, but not the quantum yield Y or the efficiency factor η).

By implementing these criteria in a differential evolution algorithm (code available on GitHub[2]), we obtained a set of parameters and results for each case (**Table S1** and **S2**, simulated traces in **Figure S13**). Note that the measured time constant of the enhanced response was likely limited by the camera response time, rather than the protein kinetics.

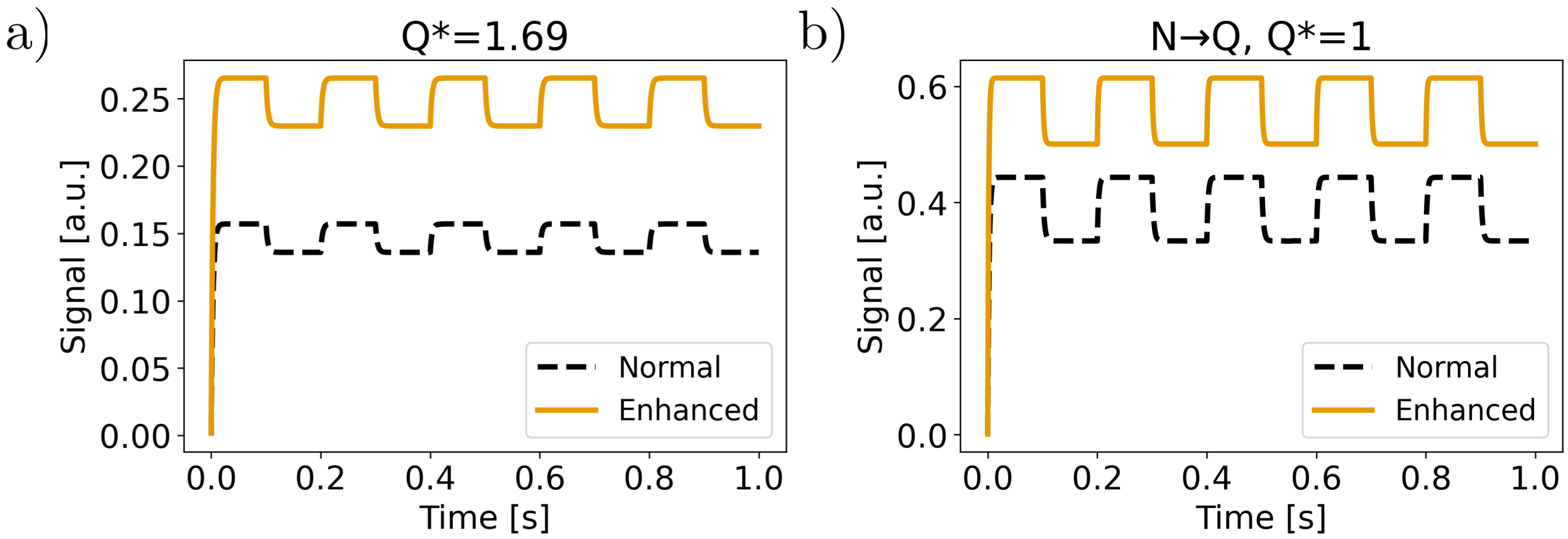

Figure S13: Numerically simulated traces for the apparent occupancy of the Q-state of QuasAr6a under the influence of a square wave in membrane potential (−70 mV to +30 mV). **a,** Plasmonic enhancement is assumed to enhance the Q-state only. **b,** Plasmonic enhancement is assumed to enhance k_3 only.

Table S1: Best parameters from genetic algorithm fitting of the experimental data.

**Parameter**		**Q***	**N →Q**	**N →Q, Q***
k_1 $[\mathrm{s}^{-1}]$		12338	91991	99755
$k_2{=}k_{2m}V + k_{2q}$	k_{2m} $[\mathrm{V}^{-1}\mathrm{s}^{-1}]$	219915	284200	206712
	k_{2q} $[\mathrm{s}^{-1}]$	30695	29085	19088
k_3 $[\mathrm{s}^{-1}]$	Control	100	252	443
	Enhanced	100	505	1184
k_4 $[\mathrm{s}^{-1}]$		261	232	119
k_5 $[\mathrm{s}^{-1}]$		9851	9800	9723
$\left\lvert\vec{E}\right\rvert^2 / \left\lvert\vec{E^0}\right\rvert^2$ [a.u.]		1.69	1	1.34

Table S2: Comparison of the features and quantification of the Huber loss for experimental and simulated conditions.

**Output**		**Observed results**	**Q***	**N →Q**	**N →Q, Q***
Brightness Enhancement [a.u.]		0.69	0.69	0.50	0.88
$\frac{\Delta F}{F}$ [a.u.]	Control	0.37	0.16	0.33	0.33
	Enhanced	0.11	0.16	0.23	0.15
k_{up} [s^{-1}]	Control	230	299	412	414
	Enhanced	1300	299	598	900
k_{down} [s^{-1}]	Control	270	303	343	250
	Enhanced	930	303	455	459
Huber loss [a.u.]			14.8	11.6	8.8

Single exponential fit for voltage sensitivity

In the experiments presented in **Figure 3**, a single response time was assumed in the fitting. That is because the effective response of a distribution of protein distances (some close to the nanostars, others farther) will still lead to a single exponential response.

When we assume that the plasmonic coupling modifies the rate of each fluorophore according to a Gaussian distribution of k, centered in k_0 having standard deviation c:

$$P(k) = \frac{1}{\sqrt{2\pi}\, c} \exp\left[-\frac{(k-k_0)^2}{2c^2}\right] \tag{S.23}$$

the ensemble-averaged fluorescence decay results:

$$I(t) = \int_0^\infty P(k) e^{-kt}\, dk = \frac{1}{2} \exp\left(-k_0 t\right) \exp\left(\frac{c^2 t^2}{2}\right) \operatorname{erfc}\left(\frac{tc^2 - k_0}{\sqrt{2}\, c}\right) \tag{S.24}$$

Since a two-exponential fit adds a poorly-determined extra component that might be misinterpreted, and the above integral shows that a continuous spread of k values still produces a near-monoexponential decay when one population dominates, we chose to report the single effective time constant $1/k_{eff}$. In practice, the single exponential fits the data with no systematic residuals, so there is no need for a multi-exponential fit.

Table S3: Statistical significance tests

Figure	Type of test	Comparison	p-value	Significance
2c	t-test (Bonferroni)	ns - Q1	0.0097	**
		ns - Q1+ns (computed)	0.0053	**
		ns - Q1+ns (measured)	0.0007	***
		Q1 - Q1+ns (computed)	1	n.s.
	Paired t-test	Q1 - Q1+ns (measured)	0.0386	*
		Q1+ns (computed) - Q1+ns (measured)	0.0478	*
2d	t-test (Bonferroni)	ns - Q6a	0.0027	**
		ns - Q6a+ns (computed)	0.0018	**
		ns - Q6a+ns (measured)	<0.0001	****
		Q6a - Q6a+ns (computed)	1	n.s.
	Paired t-test	Q6a - Q6a+ns (measured)	0.0050	**
		Q6a+ns (computed) - Q6a+ns (measured)	0.0072	**
2h	Mann-Whitney	Control - NSs	<0.0001	****
3c	t-test (Bonferroni)	Control - MPTMS	1	n.s.
		Control - 50 nm stars	1	n.s.
		Control - 90 nm spheres	0.2490	n.s.
		Control - Resonant stars	0.0012	**
		MPTMS - 50 nm stars	0.3402	n.s.
		MPTMS - 90 nm spheres	1	n.s.
		MPTMS - Resonant stars	1.2E-04	***
		50 nm stars - 90 nm spheres	0.0532	***
		50 nm stars - Resonant stars	0.0106	*
		90 nm spheres - Resonant stars	<0.0001	****
3d (up)	t-test (Bonferroni)	Control - MPTMS	1	n.s.
		Control - 50 nm stars	1	n.s.
		Control - 90 nm spheres	1	n.s.
		Control - Resonant stars	<0.0001	****
		MPTMS - 50 nm stars	1	n.s.
		MPTMS - 90 nm spheres	1	n.s.
		MPTMS - Resonant stars	<0.0001	****
		50 nm stars - 90 nm spheres	1	n.s.
		50 nm stars - Resonant stars	<0.0001	****
		90 nm spheres - Resonant stars	<0.0001	****
3d (down)	t-test (Bonferroni)	Control - MPTMS	1	n.s.
		Control - 50 nm stars	1	n.s.
		Control - 90 nm spheres	1	n.s.
		Control - Resonant stars	<0.0001	****
		MPTMS - 50 nm stars	1	n.s.
		MPTMS - 90 nm spheres	1	n.s.
		MPTMS - Resonant stars	<0.0001	****
		50 nm stars - 90 nm spheres	1	n.s.
		50 nm stars - Resonant stars	<0.0001	****
		90 nm spheres - Resonant stars	<0.0001	****
3e	Welch's t-test	Nanostars - MPTMS only	0.172	n.s.

References

(1) Fu, B.; Flynn, J. D.; Isaacoff, B. P.; Rowland, D. J.; Biteen, J. S. Super-Resolving the Distance-Dependent Plasmon-Enhanced Fluorescence of Single Dye and Fluorescent Protein Molecules. *Journal of Physical Chemistry C* **2015**, *119*, DOI: 10.1021/acs.jpcc.5b05154.

(2) GitHub repository. https://github.com/Brinkslab/plasmon_gevi.

(3) Locarno, M.; Brinks, D. Analytical calculation of plasmonic resonances in metal nanoparticles: A simple guide. *American Journal of Physics* **2023**, *91*, 538, DOI: 10.1119/5.0094967.

(4) Link, S.; El-Sayed, M. A. Spectral Properties and Relaxation Dynamics of Surface Plasmon Electronic Oscillations in Gold and Silver Nanodots and Nanorods. *Journal of Physical Chemistry B* **1999**, *103*, DOI: 10.1021/jp9917648.

(5) Novotny, L.; Hecht, B. *Principles of Nano-Optics*, 2nd ed.; 2009; Chapter 6.5, pp 188–192, DOI: 10.1017/CBO9780511794193.

(6) Shukla, R.; Bansal, V.; Chaudhary, M.; Basu, A.; Bhonde, R. R.; Sastry, M. Biocompatibility of gold nanoparticles and their endocytotic fate inside the cellular compartment: A microscopic overview. *Langmuir* **2005**, *21*, DOI: 10.1021/la0513712.

(7) Guerrero-Martínez, A.; Barbosa, S.; Pastoriza-Santos, I.; Liz-Marzán, L. M. Nanostars shine bright for you. Colloidal synthesis, properties and applications of branched metallic nanoparticles. *Current Opinion in Colloid and Interface Science* **2011**, *16*, DOI: 10.1016/j.cocis.2010.12.007.

(8) Barbosa, S.; Agrawal, A.; Rodríguez-Lorenzo, L.; Pastoriza-Santos, I.; Alvarez-Puebla, R. A.; Kornowski, A.; Weller, H.; Liz-Marzán, L. M. Tuning size and sensing properties in colloidal gold nanostars. *Langmuir* **2010**, *26*, DOI: 10.1021/la102559e.

(9) Silapetere, A. et al. QuasAr Odyssey: the origin of fluorescence and its voltage sensitivity in microbial rhodopsins. *Nature Communications* **2022**, *13*, 5501, DOI: 10.1038/s41467-022-33084-4.

(10) Kneipp, J.; Kneipp, H.; McLaughlin, M.; Brown, D.; Kneipp, K. In vivo molecular probing of cellular compartments with gold nanoparticles and nanoaggregates. *Nano Letters* **2006**, *6*, DOI: 10.1021/nl061517x.

(11) Lide, D. R. *CRC Handbook of Chemistry and Physics*, 6th ed.; CRC Press (Internet version), 2005.

(12) Hale, G. M.; Querry, M. R. Optical Constants of Water in the 200-nm to 200-μm Wavelength Region. *Applied Optics* **1973**, *12*, DOI: 10.1364/ao.12.000555.

(13) König, T. A.; Ledin, P. A.; Kerszulis, J.; Mahmoud, M. A.; El-Sayed, M. A.; Reynolds, J. R.; Tsukruk, V. V. Electrically tunable plasmonic behavior of nanocube-polymer nanomaterials induced by a redox-active electrochromic polymer. *ACS Nano* **2014**, *8*, DOI: 10.1021/nn501601e.

(14) Fu, Y.; Zhang, J.; Lakowicz, J. R. Plasmonic enhancement of single-molecule fluorescence near a silver nanoparticle. *Journal of Fluorescence* **2007**, *17*, DOI: 10.1007/s10895-007-0259-0.

(15) Tian, H. et al. Video-based pooled screening yields improved far-red genetically encoded voltage indicators. *Nature Methods* **2023**, DOI: 10.1038/s41592-022-01743-5.

(16) Kubin, R. F.; Fletcher, A. N. Fluorescence quantum yields of some rhodamine dyes. *Journal of Luminescence* **1982**, *27*, 455–462, DOI: 10.1016/0022-2313(82)90045-X.

(17) Reungpatthanaphong, P.; Dechsupa, S.; Meesungnoen, J.; Loetchutinat, C.; Mankhetkorn, S. Rhodamine B as a mitochondrial probe for measurement and monitoring of mitochondrial membrane potential in drug-sensitive and -resistant

cells. *Journal of Biochemical and Biophysical Methods* **2003**, *57*, 1–16, DOI: 10.1016/S0165-022X(03)00032-0.

(18) Moreau, D.; Lefort, C.; Burke, R.; Leveque, P.; O'Connor, R. P. Rhodamine B as an optical thermometer in cells focally exposed to infrared laser light or nanosecond pulsed electric fields. *Biomedical Optics Express* **2015**, *6*, 4105–4117, DOI: 10.1364/BOE.6.004105.
